# Interracial contact differentially shapes brain networks involved in social and non-social judgments from faces: a combination of univariate and multivariate approaches

**DOI:** 10.1093/scan/nsab090

**Published:** 2021-07-28

**Authors:** Grace Handley, Jennifer Kubota, Jasmin Cloutier

**Affiliations:** Department of Psychological and Brain Sciences, University of Delaware, Newark, DE 19716, USA; Department of Psychological and Brain Sciences, University of Delaware, Newark, DE 19716, USA; Department of Political Science and International Relations, University of Delaware, Newark, DE 19716, USA; Department of Psychological and Brain Sciences, University of Delaware, Newark, DE 19716, USA

**Keywords:** interracial contact, multivariate network analysis

## Abstract

The present work explores the relationship between interracial contact and the neural substrates of explicit social and non-social judgments about both racial ingroup and outgroup targets. Convergent evidence from univariate and multivariate partial least squares (PLS) analyses reveals that contact shapes the recruitment of brain regions involved in social cognition similarly for both ingroup and outgroup targets. Results support the hypothesis that increased contact is associated with generalized changes in social cognition toward both ingroup and outgroup faces. Specifically, regardless of target race, low- and average-contact perceivers showed the typically observed increased recruitment of temporoparietal junction and dorsomedial prefrontal cortex during social compared to perceptual judgments. However, high-contact perceivers did not show selective recruitment of these brain regions for social judgments. Complimenting univariate results, multivariate PLS analyses reveal that greater perceiver contact leads to reduced co-activation in networks of brain regions associated with face processing (e.g. fusiform gyrus) and salience detection (e.g. anterior cingulate cortex and insula). Across univariate and multivariate analyses, we found no evidence that contact differentially impacted cross-race face perception. Instead, when performing either a social or a novel perceptual task, interracial contact appears to broadly shape how perceivers engage with all faces.

Our ability to infer social information from faces facilitates our remarkable capacity for complex social interactions. Reflecting the importance of this ability, a substantial body of research has identified an extended network of brain regions involved when inferring social information from faces (Haxby *et al.*, 2000, 2001; Cloutier *et al.*, 2011b; Haxby and Gobbini, 2011; Dang *et al.*, 2019). However, as society grows increasingly diverse, there is a need to understand how interracial contact shapes social cognition. In light of the extensive literature exploring how contact influences a variety of cognitive and affective processes (e.g. Tausch and Hewstone, 2010), it is surprising that little is known about how contact shapes neural responses during fundamental social cognitive tasks. We therefore sought to investigate potential neural mechanisms by which contact may shape how people infer social *vs* non-social information from same-race and cross-race individuals. Specifically, here we extend earlier work on how childhood contact shapes brain network activity during impression formation (e.g. Cloutier *et al.*, 2017) by testing how contact across the lifespan impacts explicit mental state inferences in contrast to non-social facial judgments. This allows us to differentiate the specific impact of contact on mentalizing, a critical component of theory-of-mind and social cognition, from how it may shape neural responses to faces irrespective of task.

In addition to the accumulation of behavioral evidence that interracial contact shapes intergroup dynamics (e.g. Dovidio *et al.*, 2003; Brown and Hewstone, 2005; Pettigrew and Tropp, 2006; Tausch and Hewstone, 2010; Pettigrew *et al.*, 2011; Kubota *et al.*, 2017; Quinn *et al.*, 2019), brain imaging evidence highlights the importance of intergroup contact in shaping person perception and evaluation. Indeed, interracial contact modulates amygdala responses to outgroup faces (Telzer *et al.*, 2013; Cloutier *et al.*, 2014). This region has been shown to index the social salience of stimuli (Adolphs, 1999, 2010; Anderson and Phelps, 2001; Sander *et al.*, 2003; Adolphs and Spezio, 2006; Brosch *et al.*, 2008; Cunningham and Brosch, 2012). Specifically, Cloutier *et al.* (2014) found that greater interracial childhood contact was associated with greater reduction in amygdala activity in response to familiar (as opposed to novel) Black faces. The researchers posit that high-contact perceivers experience these familiar Black faces as less socially salient than low-contact perceivers. Similarly, Telzer *et al*. (2013) showed that adolescents who had greater peer diversity contact had decreased amygdala activity when viewing Black faces relative to their peers who reported less peer diversity contact. Together, these findings suggest that interracial contact shapes evaluative responses during face perception; however, no research to date has directly considered how contact impacts the mechanisms supporting explicit social judgments from faces.

Across many different tasks, bilateral temporoparietal junction (TPJ) and medial prefrontal cortex (MPFC) activity has been shown to increase during social cognition (for reviews, see Amodio and Frith, 2006; Schurz *et al.*, 2014). For example, bilateral TPJ is selectively recruited when participants read stories about a person’s mental states relative to when they read stories about a person’s physical characteristics (Saxe and Kanwisher, 2003). The right TPJ is believed to be uniquely selective for inferring others’ mental states as opposed to thinking about other socially relevant information about a person (e.g. Saxe and Wexler, 2005; Young *et al.*, 2010). Additionally, clinical studies implicate abnormal TPJ function with deficits in social cognitive ability. For example, individuals with autism spectrum disorders, unlike neurotypical individuals, do not show increased right TPJ activity when mentalizing compared to the performance of physical judgments (Lombardo *et al.*, 2011). Additionally, patients with TPJ damage display significant deficits in mental state representation during both story-based and video-based false belief tasks (Samson *et al.*, 2004). These studies suggest normal TPJ function is required for accurately inferring others’ mental states.

The MPFC has also been implicated in social cognition. Increased MPFC activity is observed when participants comprehend stories that require mental state attributions compared to stories that require thinking about peoples’ physical locations (Fletcher *et al.*, 1995; Gallagher *et al.*, 2000; Vogeley *et al.*, 2001). Additionally, inferences of psychological states reliably recruit MPFC to a greater extent than judgments of physical body parts (Mitchell *et al.*, 2005a). More specifically, a dorsal region of the MPFC (DMPFC) is preferentially recruited when participants judge how pleased a person’s face appears (i.e. social judgment) than when they judge a person’s facial symmetry (i.e. non-social judgment; Mitchell *et al.*, 2005b). Preferential MPFC and TPJ activity is also observed when perceivers view personally familiar faces relative to unfamiliar faces, presumably because a rich array of person-knowledge is associated with them (Gobbini *et al.*, 2004, 2007; Cloutier *et al.*, 2011b; Di Oleggio Castello *et al.*, 2017). Similarly, the MPFC and TPJ have been shown to be recruited in response to faces paired with person-knowledge violating social expectations (Cloutier *et al.*, 2011a; Ma *et al.*, 2012; Mende-Siedlecki *et al.*, 2013), presumably because greater social cognitive efforts need to be expended to form impressions of them (Hamilton and Sherman, 1996; Macrae *et al.*, 1999). Together, these findings demonstrate that much like the TPJ, the MPFC is involved in a variety of tasks involving face processing and social cognition.

In addition to the roles of TPJ and MPFC, the superior temporal sulcus (STS) is also found to support social inferences from facial cues (i.e. the eyes; Hoffman and Haxby, 2000; Cloutier *et al.*, 2008; Adams *et al.*, 2010; Schurz *et al.*, 2014), suggesting that the STS may support social cognitive response based on visual social cues. Indeed, the STS may be particularly sensitive to biological motion (Allison *et al.*, 2000; Vaina *et al.*, 2001). However, the STS has also been shown to respond to faces associated with affective behaviors relative to novel faces (Todorov *et al.*, 2007). Like MPFC and TPJ, this region is also consistently activated more strongly by personally familiar faces than other faces (Gobbini *et al.*, 2004; Leibenluft *et al.*, 2004; Di Oleggio Castello *et al.*, 2017). Together, these studies suggest that STS may have a unique role in representing social aspects related to other entities. Despite this well-characterized social cognitive brain network that includes the TPJ, MPFC and STS, no work has considered how interracial contact may influence these and other regions when perceivers explicitly make social compared to non-social inferences from faces.

Complementing previous efforts focusing on univariate analysis of brain activity in regions of interests (ROIs; i.e. the amygdala), interracial contact was recently found to modulate activity in large networks of brain regions, including those supporting face processing, salience detection and mentalizing, when perceivers were simply asked to form impressions of Black and White faces (Cloutier *et al.*, 2017). With regard to brain regions supporting social cognition (e.g. Amodio and Frith, 2006; Schurz *et al.*, 2014), contact was associated with decreased recruitment of a brain network that includes the MPFC, TPJ and STS. Similarly, in brain regions supporting salience detection (e.g. Seeley *et al.*, 2007; Menon and Uddin, 2010; Uddin, 2015), contact was associated with decreased recruitment of a brain network that includes the insula and anterior cingulate cortex (ACC). The reduced recruitment of these brain networks as a function of increased interracial contact was found irrespective of the race or familiarity of the presented face, suggesting that contact may broadly shape social cognitive mechanisms beyond intergroup contexts. The present work aimed to directly address this question by assessing brain responses when perceivers explicitly perform social compared to non-social judgments from faces. Specifically, we hypothesized that contact would lead to ‘decreased’ recruitment of brain regions involved in social cognition in a target race-generalized manner relative to a task requiring novel perceptual judgments of the same faces.

In addition to the previously presented findings (Cloutier *et al.*, 2017), this prediction is supported by several social cognitive models of intergroup face processing, proposing that experience with faces outside of those typically encountered may render faces less distinctive (e.g. Valentine, 1991; Levin, 2000; Correll *et al.*, 2017). Indeed, experience with a variety of faces may change both the central tendency and the normal degree of variation of perceived faces. Additionally, motivational factors resulting from experience or task demands may also contribute to minimize the discrepancy between encountered faces and a face reference (Correll *et al.*, 2017). This reduction in perceived distinctiveness or saliency of encountered faces may in turn decrease the need to spontaneously individuate them, irrespective of race. This in turn can lead to the prediction that increased experience with a variety of faces may actually decrease spontaneous social cognitive engagement. Further supporting this possibility, we found that high-contact White perceivers are less accurate than low-contact White perceivers at inferring complex mental states from Black and White target faces in the Reading the Mind in the Eyes task, perhaps reflecting lower motivational salience of these targets at baseline; however, motivation to attend to the task reversed this effect (Handley *et al.*, in press). Thus, high-contact perceivers may not engage as effortfully as low-contact perceivers with faces when navigating their social worlds on an everyday basis.

This study represents the first attempt to directly investigate how interracial contact shapes neural processes supporting social versus non-social judgments from faces. It also importantly illuminates how social and non-social processes may be affected by individual differences among perceivers, a central question underlying modern social neuroscience (Stanley and Adolphs, 2013). We employ both univariate confirmatory ROI-based analyses, exploratory whole-brain GLM analyses and a data-driven multivariate network approach using task and behavioral partial least squares (PLS) analyses. PLS is a data-driven multivariate technique that aims to identify significant latent variables (LVs) that explain relationships between brain network activity and experimental variables of interest (McIntosh and Lobaugh, 2004; Krishnan *et al.*, 2011). In this study, we use ‘task PLS’ to identify LVs that maximally explain covariance between blood-oxygen-level-dependent (BOLD) activity across multiple voxels and the race of target faces (Black *vs* White) while performing the two tasks (mentalizing *vs* non-mentalizing). We then use ‘behavioral PLS’ to identify LVs that explain covariance as a function of individual differences in lifetime contact and patterns of neural activity associated with these experimental conditions. By combining univariate and multivariate analysis approaches, we can both interpret our findings in the context of previous univariate studies contrasting social and non-social judgments from faces and extend our understanding of how individual differences in lifetime interracial contact broadly affect recruitment of networks involved in social cognition and salience detection (Cloutier *et al.*, 2017).

## Study overview

Perceivers varying in interracial contact were asked to either perform a social judgment (‘how interested is this person in the experiment?’) or to perform a non-social judgment (‘how symmetrical is this person’s face?’; Mitchell *et al.*, 2005b) from Black or White targets. We tested whether individual differences in lifetime interracial contact were associated with differential activity in brain regions previously implicated in social salience processing (amygdala; Adolphs, 1999, 2010; Sander *et al.*, 2003; Adolphs and Spezio, 2006; Brosch *et al.*, 2008; Cunningham and Brosch, 2012) and social cognition [bilateral TPJ, dorsomedial prefrontal cortex (DMPFC) and bilateral STS; Saxe and Kanwisher, 2003; Mitchell *et al.*, 2005b; Amodio and Frith, 2006; Spreng *et al.*, 2009; Schurz *et al.*, 2014].

**Hypotheses.** We predicted that interracial contact would shape brain activity in these ROIs irrespective of target race. Based on previous findings that contact was associated with decreased recruitment of regions supporting social cognition during private impression formation (Cloutier *et al.*, 2017), we predicted that contact would be associated with relative decreased activity in bilateral TPJ, DMPFC and bilateral STS during the social task compared to the non-social task. Given the amygdala’s role in detecting social and motivational salience, we predicted that when performing a social judgment, perceivers with greater interracial contact may find Black targets less salient and consequently display less amygdala activity compared to those with less interracial contact.

We also planned to use whole-brain GLM and PLS network analyses in an exploratory manner; however, consistent with recommendations for best practices in analyzing functional magnetic resonance imaging (fMRI) data (e.g. Zandbelt *et al.*, 2008; Vul and Pashler, 2017), we emphasize our confirmatory ROI-based analyses about which we had a priori hypotheses.

## Methods

### Participants

We scanned sixty-one White participants (*M*_age_ = 25.05, s.d. = 7.34, 28 females, 31 males, 2 other gender) recruited from the University of Chicago and from the surrounding community. All participants were neurotypical, right-handed, proficient English speakers with normal or corrected-to-normal vision. They were not colorblind, had no history of drug use, had no prior head injuries, did not take psychotropic medications and did not have any chronic illness affecting their mental, neural or autonomic function. According to our a priori inclusion criteria, all participants were of White European-American (non-Hispanic or mixed race) descent, between the ages of 18 and 50 years, and were born in the USA. Participants were prescreened to ensure that they met these eligibility criteria. To ensure an adequate distribution of contact, we also screened for contact using an abbreviated version of the contact questionnaire described later. Twenty participants (32.8%) were screened to have a minimum of 15% childhood contact with Black people. The remaining participants could report any level of contact.

#### Data exclusions

Data from three participants were excluded due to technical scanner issues rendering data unusable, and data from another four participants were excluded due to excessive movement during the scan (>3 mm). Our final sample included 54 participants (*M*_age_ = 24.31, s.d. = 6.47, 27 females, 25 males, 2 other gender).

### Stimuli

All stimuli were sourced from a pool of 372 Black and White faces from the Chicago Face Database (Ma *et al.*, 2015). The final stimulus set included 60 unique faces (all male, 30 Black). All faces showed direct eye gaze and upright head position, and none wore glasses or piercings. All images were equated on contrast and luminance using the SHINE toolbox (Willenbockel *et al.*, 2010). Images were cropped to be presented centrally on a 504 × 632-pixel frame and backgrounds were changed to light gray. Overall, 97.1% of the final 60 faces in the stimulus set were correctly identified as either Black or White. Stimuli were also equated on emotional expression, with equal proportions of Black and White faces being rated as angry, happy, neutral or sad (overall: 13.7%, 12.4%, 59.9% and 14.0%, respectively). Expression intensity also did not differ between the Black and White faces (overall *M *= 5.58 on a scale from 5 to 9). Trustworthiness, dominance, attractiveness, likability and threat judgments were rated on a 7-point scale (e.g. 1 = not trustworthy at all and 7 = very trustworthy). Mean trustworthiness (*M *= 3.08), dominance (*M *= 4.02), attractiveness (*M* = 2.87) and likability (*M *= 3.39) did not differ between the Black and White faces. For additional details on the equating procedures, see Mattan *et al.* (2018a).

### Experimental protocol

We assessed brain activity during social judgments from faces using a modified version of a task developed by Mitchell and colleagues (Mitchell *et al.*, 2005b). In the original task, participants rated either how pleased a person looked about having their photograph taken (the social task) or how symmetrical their face seemed (the non-social judgment). This task used White stimuli only. We slightly modified Mitchell and colleagues’ task and prompted participants to instead think about how interested the person looked in completing an experiment. We made this change in order to avoid valenced connotations associated with appearing pleased. We did not change the non-social (facial symmetry rating) judgment. In this modified version of the task, we included both White and Black stimuli, described above.

Prior to their scan, participants completed a number of surveys including an interracial contact questionnaire and various unrelated questionnaires used for resting state analyses and another fMRI task completed after the mentalizing/non-mentalizing task (for a complete list of all questionnaires see Supplementary Discussion 1). Immediately before their scanning session, participants completed a training procedure to familiarize themselves with the task, rating scale, use of the button box, and response cue.

During the scanning procedure, all 60 faces were rated twice: once when performing the social judgments (cue: ‘How interested are these people in the experiment?’) and once when performing the non-social judgments (cue: ‘How symmetrical are these faces?’; henceforth these judgment blocks are referred to as the social task and the non-social task, respectively). Stimuli were presented over four runs. Each run always included one block of social judgments and one block of non-social judgments. The presentation order of these blocks was counterbalanced across runs and participants. Participants made their ratings in blocks of 15 trials each, with seven or eight trials of each race per block (alternated so that there were ultimately 30 Black-non-social trials, 30 White-non-social trials, 30 Black-social trials and 30 White-social trials); in each block participants rated either interest (social) or symmetry (non-social). Task order was counterbalanced across participants. Within each block, stimuli were presented in a rapid-event-related manner with Black and White trials randomly intermixed (with no more than three Black or White trials in a row). Stimulus presentation was optimized through optseq2 (available at http://surfer.nmr.mgh.harvard.edu/optseq; see Supplementary Discussion 2). Participants gave their ratings on a four-point scale using a button box that they were trained to use prior to their scan.

### Interracial contact questionnaire

Participants completed an online questionnaire that assessed the composition of their childhood and current social networks across racial groups (Asian, Black, Hispanic, White and other; Cloutier *et al.*, 2014). This questionnaire asked participants to report their personal familiarity with outgroup members across several social categories varying in closeness (e.g. friendships, peers, neighbors, etc.) during different stages of their life (0–6 years old, 7–12 years old, 13–18 years old, and currently). Participants answered questions about each life stage separately (i.e. they answered all of the questions about the 0–6 year old stage first, then the 7–12-year-old stage, etc.). Each life stage included questions such as, ‘What percentage of your neighbors (think about the closest 100 households) belonged to each of the following categories?’ and ‘Think about the people^1^ you knew on a first name basis (neighbors, teammates, classmates, etc.). What percentage belonged to each of the following categories?’ Participants were instructed that their responses for each question must add up to 100%.

Each participant’s average childhood and current contact with Black and White people were calculated, respectively, and a difference score between contacts with Black *vs* White people was computed as their average contact with White people subtracted from their average contact with Black people. Thus, each participant had separate childhood and current contact scores that ranged from −100 (0% contact with Black people) to +100 (100% contact with Black people). As childhood and current contact were moderately correlated [*r*(52) = 0.316, *P* = 0.020, 95% CI = (0.052, 0.538)], we computed a composite contact score to index participants’ lifetime interracial contact. Specifically, we calculated a measure of lifetime contact by averaging each participant’s childhood and current contact difference scores, which we used for all reported analyses. Our sample had a mean lifetime contact score of −57.14 with a standard deviation of 18.45. In other words, our sample participants had relatively more contact with White than Black individuals in their social networks. We present several follow-up analyses to decompose interactions using this measure below with contact centered at −2 s.d. values below the mean (i.e. low contact, centered at −94.04) and +2 s.d. values above the mean (i.e. high contact, centered at −20.24). For all confirmatory ROI analyses using childhood and current contact separately as predictors, see Supplementary Discussion 3.

### ROIs

Based on previous research, we selected bilateral TPJ, DMPFC and bilateral STS as primary ROIs (Spreng *et al.*, 2009; Cloutier *et al.*, 2017). Coordinates for TPJ, DMPFC and STS were selected from a comprehensive meta-analysis by Spreng *et al.* (2009). We also selected the amygdala as an ROI based on previous work showing that childhood contact is associated with changes in the amygdala activity when viewing faces (Cloutier *et al.*, 2014).

### fMRI data acquisition

The fMRI session lasted approximately 18 min (each of the four runs lasted 274 s). Faces were presented for 4.0 s followed immediately by a 2.0 s response window during which a green fixation cross was displayed that cued participants to rate the faces. Between trials, participants viewed a fixation cross for jittered intervals lasting between 1.0 and 7.0 s. Anatomical and functional imaging was performed on a 3T Philips Achieva Quasar scanner at the University of Chicago Magnetic Resonance Imaging Research Center. Functional images were collected in four functional runs of 137 TRs (repetition time (TR) = 2.0 s) each, using pulse sequence parameters (TR/echo time = 2000/25 ms, flip angle = 79°, contiguous slices with 3.28 mm thickness, gap = 0.72 mm, field-of-view (FOV) = 210 × 210 mm, approximately 64 × 64 mm matrix, 3.28 × 3.28 mm^2^ voxel size). High-resolution structural images were acquired in the sagittal plane using a T1-weighted 3D Turbo Field Echo (TFE/Magnetization-Prepared Rapid Gradient-Echo (MP-RAGE)) anatomical scan (TR = 8.5 ms, echo time = 4.0 ms, FOV = 240 × 228 mm, 1.0 mm slice thickness, no gap, 240 × 228 mm matrix, 1.0 × 1.0 × 1.0 mm^3^ voxel size). Functional imaging data were preprocessed using SPM8 (https://www.fil.ion.ucl.ac.uk/spm), facilitated by a custom suite of scripts for fMRI analysis (https://github.com/ddwagner/SPM8w), to remove sources of noise and artifacts and realigned within and across runs to correct for head movement and transformed into a standard anatomical space (3 mm isotropic voxels) based on the ICBM 152 brain template (MNI, Montreal Neurological Institute) which approximates the Talairach and Tournoux atlas space (Talairach and Tournoux, 1988). Normalized data were then spatially smoothed (8 mm Full Width at Half Maximum (FWHM)) using a Gaussian Kernel to increase the signal-to-noise ratio and reduce the impact of anatomical variability not corrected for by stereotaxic normalization.

For each participant, GLMs were constructed to examine condition-specific brain activity as a function of the task (social or non-social) and target race (Black or White). GLMs incorporating each of the four conditions and covariates of non-interest (a session mean, a linear trend to account for low-frequency drift and six movement parameters derived from realignment corrections) were convolved with a canonical hemodynamic response function and used to compute parameter estimates (β) for each condition at each voxel. At level 2, *z*-scored lifetime contact (Black – White) was included as a between-subjects factor for whole-brain exploratory analyses.

### Data analysis

We used mixed-effects regression to analyze both the behavioral and fMRI ROI data with the lme4 package (Bates *et al.*, 2014) in the R programming language (R Core Team, 2016). All statistical tests were two-tailed. The within-subjects factors were target race (−0.5 = Black faces and 0.5 = White faces) and task (−0.5 = non-mentalizing and 0.5 = mentalizing). The between-subjects factor was lifetime contact, which was converted to a *z*-score. For all fMRI results, coordinates are given in MNI space.

#### Behavioral

The dependent variable was rated interest or symmetry (1 = least interested or symmetrical to 4 = most interested or symmetrical). Half of the participants were instructed to make their ratings in a descending order (1 = most interested or symmetrical to 4 = least interested or symmetrical); these responses were reverse coded prior to analysis. On trials where participants did not select a response, their responses were coded as missing data; these responses were removed from the dataset prior to behavioral data analysis (406 out of 7320 trials, 5.55%). These trials were not excluded from any subsequent analysis of fMRI data. We allowed for between-subjects variance in intercepts and slopes as a function of target race and task (i.e. random effects) to account for participant variations in response as a function of target race and task. The behavioral results from this task were of minimal theoretical interest for the present work; we therefore present these results in Supplementary Discussion 5.

#### ROI analyses

We analyzed BOLD activity in a priori ROIs (bilateral TPJ, DMPFC, bilateral STS and bilateral amygdala) as a function of target race, task and participants’ lifetime contact scores. We attempted to model as many random effects as possible without overfitting data. In the event of convergence failures or model overfitting, we followed a uniform procedure for the simplification of random-effects structures (Bates *et al.*, 2018). For the ROI analyses we were only able to model random effects for the intercept because of trial numbers per condition.

#### Exploratory whole-brain analyses

In addition to the ROI analyses, we ran two exploratory whole-brain GLM analyses to test the effect of lifetime contact (*z*-scored) on our primary contrasts of interest: social > non-social and Black > White. We used an uncorrected voxel-level threshold of *P* < 0.001 and a cluster extent threshold of 52 voxels as determined by AlphaSim. These results are exploratory in nature and should be interpreted in the context of the a priori ROI analyses.

#### Partial least squares analyses

We used the same analysis procedure reported by Cloutier *et al.* (2017) for both task PLS and behavioral PLS network analyses (see Cloutier *et al.*, 2017 for a detailed description of these analyses). PLS analyses were implemented using publicly available software (https://www.rotman-baycrest.on.ca/index.php?section=84) and a PLS analysis toolbox (http://web.mit.edu/seven/src/PLS/Plscmd/pls_analysis.m).

##### Task PLS analysis.

We tested the significance of each LV using a set of 2000 bootstrap samples that resampled subjects with replacement within each condition (Cloutier *et al.*, 2017; Mattan *et al.*, 2018b). This analysis yielded a bootstrap ratio (BSR) for each voxel that accounts for how reliably that voxel contributes to the LV. In other words, the BSR values provide a measure of how reliable a voxel’s contribution is to a given spatial pattern (McIntosh and Lobaugh, 2004). These BSR values are used to index the reliability of experimental effects and are not statistical tests; therefore, corrections for multiple comparisons are unnecessary (McIntosh and Lobaugh, 2004). BSRs were then mapped on brain images. BSRs were thresholded at the 95% confidence interval, which corresponds to voxels with BSRs above +2.5 or below −2.5. We used xjview (http://www.alivelearn.net/xjview) to identify contiguous clusters containing at least 20 voxels with BSRs that satisfied this threshold requirement.

##### Behavioral PLS.

As in the task PLS analysis, we ran 2000 bootstrap samples resampling subjects with replacement within each condition, conserving each participant’s contact score. We used 95% confidence intervals to test the reliability of brain-contact score correlations specific to each condition for each significant LV. We again used xjview (http://www.alivelearn.net/xjview) to identify contiguous clusters of at least 20 voxels containing BSRs above +2.5 or below −2.5.

## Results

### ROI analyses

#### Main effect of task in all ROIs

In all ROIs (left TPJ MNI_x, y, z_ = −56, −55, 16, 8 mm sphere; right TPJ MNI_x, y, z_ = 54, −51, 17, 8 mm sphere; DMPFC MNI_x, y, z_ = −3, 55, 23, 8 mm sphere; left STS MNI_x, y, z_ = −59, −15, −16, 8 mm sphere; right STS MNI_x, y, z_ = 57, −10, −20, 8 mm sphere; left amygdala MNI_x, y, z_ = −24, −6, −24, 4 mm sphere; right amygdala MNI_x, y, z_ = 18, −6, −21, 4 mm sphere) there was a significant main effect of task such that the social task was associated with greater activity than the non-social task (Table 1).

**Table 1.** ROI analysis results

Predictors	*B*	SE	df	95% CI	*t-*value	*P*-value
*L. TPJ*						
(Intercept)	−1.095	0.117	52	[−1.324, −0.865]	−9.352	<0.001*
Target race	0.046	0.103	156	[−0.156, 0.247]	0.444	0.658
Task	0.641	0.103	156	[0.440, 0.843]	6.241	<0.001*
Lifetime contact	0.057	0.117	52	[−0.173, 0.287]	0.487	0.629
Target race × task	−0.107	0.206	156	[−0.510, 0.296]	−0.519	0.605
Target race × lifetime contact	−0.009	0.103	156	[−0.211, 0.193]	−0.089	0.929
Task × lifetime contact	−0.104	0.103	156	[−0.306, 0.098]	−1.009	0.314
Target race × task × lifetime contact	0.045	0.206	156	[−0.359, 0.449]	0.217	0.828
*R. TPJ*						
(Intercept)	−0.848	0.097	52	[−1.038, −0.659]	−8.786	<0.001*
Target race	−0.030	0.087	156	[−0.201, 0.142]	−0.338	0.736
Task	0.778	0.087	156	[0.607, 0.949]	8.908	<0.001*
Lifetime contact	0.070	0.097	52	[−0.120, 0.259]	0.719	0.475
Target race × task	−0.004	0.175	156	[−0.347, 0.338]	−0.024	0.981
Target race × lifetime contact	−0.010	0.088	156	[−0.182, 0.161]	−0.118	0.907
Task × lifetime contact	−0.215	0.088	156	[−0.386, −0.043]	−2.451	0.015*
Target race × task × lifetime contact	0.073	0.175	156	[−0.270, 0.417]	0.419	0.676
*DMPFC*						
(Intercept)	−0.653	0.167	52	[−0.979, −0.327]	−3.921	<0.001*
Target race	−0.326	0.156	156	[−0.633, −0.020]	−2.086	0.039*
Task	0.546	0.156	156	[0.240, 0.853]	3.494	0.001*
Lifetime contact	0.078	0.167	52	[−0.249, 0.406]	0.470	0.641
Target race × task	−0.281	0.313	156	[−0.894, 0.332]	−0.897	0.371
Target race × lifetime contact	−0.025	0.157	156	[−0.333, 0.282]	−0.162	0.872
Task × lifetime contact	−0.505	0.157	156	[−0.812, −0.197]	−3.219	0.002*
Target race × task × lifetime contact	−0.088	0.313	156	[−0.703, 0.526]	−0.282	0.778
*L. STS*						
(Intercept)	−0.913	0.091	52	[−1.092, −0.734]	−9.993	<0.001*
Target race	−0.072	0.100	156	[−0.268, 0.123]	−0.728	0.468
Task	0.423	0.100	156	[0.228, 0.618]	4.250	<0.001*
Lifetime contact	−0.120	0.092	52	[−0.299, 0.060]	−1.307	0.197
Target race × task	−0.350	0.199	156	[−0.740, 0.040]	−1.760	0.080
Target race × lifetime contact	0.004	0.100	156	[−0.191, 0.200]	0.042	0.967
Task × lifetime contact	−0.132	0.100	156	[−0.327, 0.064]	−1.321	0.188
Target race × task × lifetime contact	−0.039	0.200	156	[−0.430, 0.352]	−0.197	0.844
*R. STS*						
(Intercept)	−1.060	0.088	52	[−1.232, −0.888]	−12.074	<0.001*
Target race	0.005	0.105	156	[−0.202, 0.211]	0.043	0.966
Task	0.659	0.105	156	[0.452, 0.865]	6.259	<0.001*
Lifetime contact	0.095	0.088	52	[−0.077, 0.268]	1.082	0.284
Target race × task	−0.187	0.210	156	[−0.599, 0.225]	−0.889	0.376
Target race × lifetime contact	−0.044	0.105	156	[−0.251, 0.163]	−0.417	0.677
Task × lifetime contact	−0.190	0.105	156	[−0.397, 0.017]	−1.802	0.073
Target race × task × lifetime contact	−0.182	0.211	156	[−0.595, 0.231]	−0.863	0.389
*L. amygdala*						
(Intercept)	0.074	0.109	52	[−0.140, 0.288]	0.679	0.500
Target race	0.017	0.111	156	[−0.201, 0.234]	0.151	0.880
Task	0.302	0.110	156	[0.085, 0.519]	2.722	0.007*
Lifetime contact	0.027	0.110	52	[−0.188, 0.242]	0.245	0.808
Target race × task	0.128	0.222	156	[−0.307, 0.562]	0.575	0.566
Target race × lifetime contact	−0.009	0.111	156	[−0.227, 0.208]	−0.085	0.932
Task × lifetime contact	−0.386	0.111	156	[−0.604, −0.168]	−3.475	<0.001*
Target race × task × lifetime contact	0.162	0.222	156	[−0.274, 0.598]	0.729	0.467
*R. amygdala*						
(Intercept)	0.522	0.126	52	[0.275, 0.770]	4.134	<0.001*
Target race	0.028	0.124	156	[−0.215, 0.271]	0.225	0.822
Task	0.257	0.124	156	[0.014, 0.500]	2.072	0.040*
Lifetime contact	−0.111	0.127	52	[−0.359, 0.137]	−0.875	0.386
Target race × task	0.134	0.248	156	[−0.352, 0.620]	0.539	0.590
Target race × lifetime contact	−0.121	0.124	156	[−0.365, 0.122]	−0.977	0.330
Task × lifetime contact	−0.084	0.124	156	[−0.328, 0.159]	−0.677	0.500
Target race × task × lifetime contact	0.027	0.248	156	[−0.460, 0.514]	0.110	0.912

L = left and R = right. Significant results are marked with an asterisk, *P *< 0.05.

#### Task by lifetime contact interaction in right TPJ, DMPFC and left amygdala

**Fig. 1.**
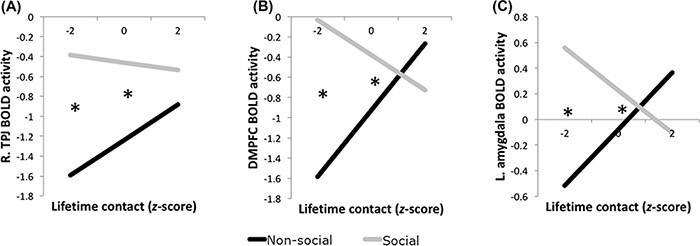
The interaction between lifetime contact (Black – White) and task significantly predicted BOLD activity in the right TPJ (A), DMPFC (B) and left amygdala (C). In all three ROIs, lower and average contact was associated with significantly greater activity during social trials than non-social trials, whereas high-contact participants did not significantly differ between social and non-social trials. Asterisks denote significant differences between conditions, *P *< 0.05.

There was a significant Task (social or non-social) × Lifetime Contact interaction in the right TPJ, DMPFC and left amygdala (Table 1). To decompose this interaction, we tested simple differences between conditions at low (−2 s.d.), average (0 s.d.) and high (+2 s.d.) lifetime contact.^2^ In all three regions, low- and average-contact participants showed significantly more activity during the social task than the non-social task, whereas high-contact participants did not significantly differ on their activity during the tasks Table 2; see also Figure 1A-C). Simple slope analyses with task dummy-coded were not significant in right TPJ, DMPFC or left amygdala.^3^

**Table 2.** Simple differences between BOLD activity during the interest task (social = 0.5) and the symmetry task (non-social = −0.5) for low-, average- and high-contact participants in three ROIsL = left and R = right. Significant results are marked with an asterisk, *p *< 0.05

	*B*	SE	*Df*	95% CI	*t*-value	*P*-value
*R. TPJ*						
Low contact	1.208	0.196	156	[0.824, 1.591]	6.170	<0.001*
Average contact	0.778	0.087	156	[0.607, 0.949]	8.908	<0.001*
High contact	0.349	0.196	156	[0.035, 0.732]	1.783	0.077
*DMPFC*						
Low contact	1.556	0.350	156	[0.869, 2.242]	4.440	<0.001*
Average contact	0.456	0.156	156	[0.240, 0.853]	3.494	0.001*
High contact	−0.463	0.350	156	[−1.149, 0.224]	−1.321	0.188
*L. amygdala*						
Low contact	1.075	0.248	156	[0.588, 1.562]	4.325	<0.001*
Average contact	0.302	0.111	156	[0.085, 0.519]	2.722	0.007*
High contact	−0.471	0.248	156	[−0.958, 0.016]	−1.894	0.060

L = left and R = right. Significant results are marked with an asterisk, *P *< 0.05.

Individuals with low (−2 s.d.) and average (0 s.d.) contact showed increased activity in these regions when performing social compared to the non-social judgments about faces, whereas high-contact individuals did not differ significantly in their recruitment of these regions. In addition, effects were similar for both ingroup and outgroup members. In other words, only as lifetime contact decreased did the right TPJ, DMPFC and left amygdala show the typical preferential response during the social trials compared to the non-social trials. Importantly, for all perceivers, activity in these brain regions during both tasks did not differ as a function of target race.

#### Main effect of target race in DMPFC

In DMPFC only, there was a significant main effect of target race. Overall DMPFC activity while rating Black faces was greater than DMPFC activity while rating White faces (Table 1). Target race did not interact with lifetime contact or task (Table 1).

### Exploratory whole-brain analyses

#### Task contrast with decreasing lifetime contact

Exploratory whole-brain analysis also confirmed the impact of lifetime contact on activity in brain regions ostensibly involved in social cognition. More specifically, consistent with results from the ROIs, we found clusters extending into the left TPJ and DMPFC that showed greater activity during social trials than non-social trials among perceivers with reduced lifetime contact (Table 3).

**Table 3.** Summary of whole-brain analysis results for the social > non-social contrast as a function of decreasing lifetime contact (*z*-scored). Uncorrected voxel-level threshold: *P *< 0.001. Extent threshold: 52 voxels

					MNI coordinates		
Brain region			*K*	*t*-value	*x*	*Y*	*z*
R. middle frontal gyrus			1808	5.28	27	27	39
	L. middle/dorsal cingulate gyrus			5.20	−12	−12	36
	R. middle/dorsal cingulate gyrus			5.19	6	12	33
	DMPFC			4.33	12	39	39
R. lingual gyrus			323	5.12	42	−81	6
	R. posterior middle temporal gyrus			4.82	48	−75	9
Cerebellum			1474	4.90	−30	−69	−45
				4.80	−15	−57	−21
				4.56	−12	−75	−36
L. TPJ			179	4.88	−45	−36	30
R. insula			469	4.76	33	21	6
	R. caudate nucleus/striatum			4.58	18	9	12
	R. insula			4.45	30	18	−3
L. superior temporal gyrus/post-central gyrus			169	4.75	−60	3	12
L. visual association area/occipital cortex			182	4.61	−33	−93	0
	L. lingual gyrus			3.74	−33	−87	12
R. parahippocampal gyrus			78	4.48	39	−18	−30
L. thalamus			151	4.45	−18	6	15
	Pallidum			4.36	−15	9	3
	Caudate nucleus			4.02	−15	24	3
R. superior parietal lobule			82	3.87	24	−63	66

#### Race contrast with decreasing lifetime contact

Consistent with the lack of significant interaction between race and lifetime contact in the ROI analyses, no clusters above threshold were found for the Black > White contrast irrespective of whether the second-level GLM accounted for decreasing lifetime contact or not.

### PLS network analyses

#### Brain networks involved in mentalizing: Task PLS

**Fig. 2.**
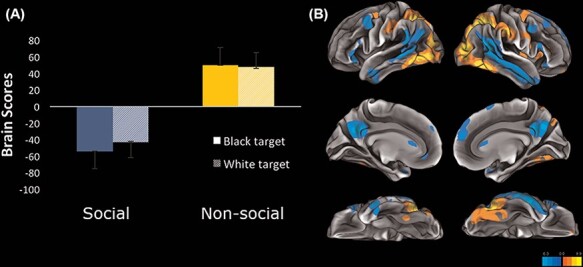
(A) The bar graph shows task PLS brain scores as a function of task (interest and symmetry) and target race (Black and White). The interest (social) task reliably contributes to explaining co-activation in the negative (blue) brain network, whereas the symmetry (non-social) task reliably contributes to explaining co-activation in the positive (yellow) brain network. (B) Visualization of brain networks involved in the social and non-social tasks. The surfaces on the left depict the left hemisphere (ascending to descending: lateral, medial and ventral surfaces) and the surfaces on the right depict the right hemisphere (ascending to descending: lateral, medial and ventral surfaces).

Task PLS revealed one significant LV (*P *< 0.001) which explained 83.571% of the crossblock covariance (see Figure 2A). During the social task, participants showed increased co-activation in a number of brain regions, including several regions typically associated with social cognition, e.g. TPJ, DMPFC, STS and precuneus (see Figure 2B and Supplementary Table S4). Contrasting this, during the non-social task participants showed increased co-activation in a number of different brain regions, including regions typically associated with visual processing, e.g. extensive bilateral occipital activation and fusiform gyrus (see Figure 2B and Supplementary Table S4). The differentially activated brain networks associated with each task confirms that the social judgment task recruited the expected social cognition network, whereas the symmetry-rating task recruited a brain network preferentially involved in visual processing.

#### Behavioral PLS

Behavioral PLS revealed two LVs that both significantly accounted for covariance between patterns of brain activity that differed across tasks as a function of participants’ lifetime contact.

##### First latent variable.

**Fig. 3.**
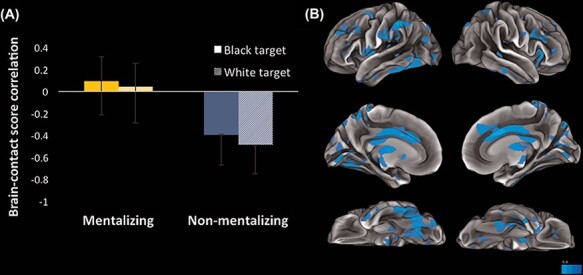
(A) This graph plots the correlations between brain scores and lifetime contact as a function of task (interest and symmetry) and target race (Black and White) for the first significant LV. The interest (social) task did not contribute to this LV, as indicated by error bars that include zero. Instead, this LV was driven by the symmetry (non-social) condition. (B) Visualization of brain networks that vary as a function of contact during the symmetry task only. Because the brain-contact score correlations are negative and the network is negative, we can interpret this network as showing increased co-activation with increasing contact. The surfaces on the left depict the left hemisphere (ascending to descending: lateral, medial and ventral surfaces) and the surfaces on the right depict the right hemisphere (ascending to descending: lateral, medial and ventral surfaces).

The first significant LV explained 58.710% of the crossblock covariance (*P *< 0.001; see Figure 3A). As lifetime contact increased, participants showed greater co-activation in a brain network recruited during the perceptual non-social task (rating symmetry); no other significant network emerged (see Figure 3B and Supplementary Table S5). This network was distinct from the social network identified in the task PLS analysis and instead included regions involved in face processing (e.g. bilateral fusiform gyrus) and salience detection (e.g. ACC and bilateral insula; Seeley *et al.*, 2007; Menon and Uddin, 2010; Uddin, 2015). Importantly, no differences associated with target race emerged.

##### Second latent variable.

**Fig. 4.**
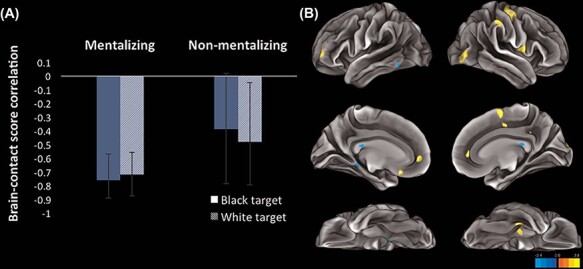
(A) This graph plots the correlations between brain scores and lifetime contact as a function of task (interest and symmetry) and target race (Black and White) for the second significant LV. All conditions reliably contributed to this LV. (B) Visualization of brain networks that vary as a function of contact. For this LV, we have both positive (yellow) and negative (blue) networks. Given the negative brain-contact score correlations, we can interpret this to mean that the positive network (yellow) showed decreased co-activation with increasing contact whereas the negative network (blue) showed increased co-activation with increasing contact. The surfaces on the left depict the left hemisphere (ascending to descending: lateral, medial and ventral surfaces) and the surfaces on the right depict the right hemisphere (ascending to descending: lateral, medial and ventral surfaces).

The second significant LV (*P* = 0.008) explained 35.093% of the crossblock covariance (see Figure 4A). As lifetime contact increased, participants showed greater co-activation in a brain network (i.e. the blue network depicted in Figure 4B) and decreased co-activation in another brain network (i.e. the yellow network depicted in Figure 4B). Although the brain networks associated with this second LV were much more restricted than those associated with the first LV, these results suggest that higher contact may be associated with generally ‘lower’ co-activation in brain regions ostensibly associated with cognitive control/effort and attention (e.g. MPFC) and inferred value (e.g. orbitofrontal cortex) during all trials (see Figure 4B and Supplementary Table S6; the yellow network in Figure 4B indicates lower co-activation with increased contact because of the negative directionality of the brain-contact score correlations in the bar graph in Figure 4A).

## Discussion

In this study exploring the impact of interracial contact on the neural substrates of explicit social and non-social judgments, we found that contact influences how we infer social information from faces irrespective of race. Specifically, contact influenced the recruitment of brain regions involved in social cognition and salience detection in a manner consistent with the hypothesis that contact increases face processing efficiency and decreases the social saliency of faces (Cloutier *et al.*, 2017). In other words, greater contact with a broader range of faces results in perceiving them as similarly salient and as less deviant from an average face irrespective of race. This greater exposure to faces varying in race should therefore allow individuals to process all kinds of faces more efficiently (Correll *et al.*, 2017). As a downstream consequence of the reduced social salience of encountered faces, high-contact individuals also show reduced recruitment of brain regions involved in social cognition during social relative to non-social tasks.

These results converge well with previously reported effects of contact on activity of extended brain network supporting face perception (Cloutier *et al.*, 2017b). Confirmatory ROI analyses revealed that increased contact was associated with relative decreases in selective recruitment of brain regions supporting social cognition (right TPJ and DMPFC) and social salience detection (left amygdala). Indeed, as expected based on previous research (Cloutier *et al.*, 2017), we found that low- and average-contact perceivers showed the expected increase in BOLD activity during the social compared with the non-social task in brain regions implicated in social cognition (right TPJ and DMPFC) and social salience (left amygdala). In comparison, high-contact perceivers did not show significantly different levels of BOLD activity during these tasks in these same regions. Importantly, and consistent with findings from Cloutier *et al.* (2017), these effects did not vary as a function of the race of the face, suggesting that contact impacts how people infer social information from faces of both outgroup ‘and’ ingroup members. This comports well with the possibility that, among White perceivers, increased contact with Black individuals is associated with reduced face saliency and increased efficiency in social inferences from both Black and White faces (i.e. their face reference is more inclusive and variations from this reference are less distinctive; Valentine, 1991; Levin, 2000; Correll *et al.*, 2017). Furthermore, reinforcing the hypothesis that greater contact reduces both outgroup and ingroup face saliency, we found that increased contact was associated with decreased preferential activity in left amygdala during the social task in response to both Black and White targets (Adolphs, 1999, 2010; Sander *et al.*, 2003; Adolphs and Spezio, 2006; Brosch *et al.*, 2008; Cunningham and Brosch, 2012).

We focus the interpretation of our results on our confirmatory ROIs in accordance with recommended best practices for fMRI data analysis (e.g. Zandbelt *et al.*, 2008; Vul and Pashler, 2017); however, overall, the results of both the exploratory whole-brain analyses and the brain network analyses converge well with our a priori hypotheses. Converging findings from the exploratory whole-brain results revealed that when inferring social information from faces (relative to a non-social task), lower-contact participants showed greater brain activity in DMPFC and left TPJ, among other regions. The differential involvement of these regions, which are considered to be part of an extended network of brain region supporting face processing (Haxby *et al.*, 2000, 2001; Cloutier *et al.*, 2011b; Haxby and Gobbini, 2011; Dang *et al.*, 2019), confirms that greater engagement is required by low- and average-contact perceivers than by high-contact perceivers during social judgments from faces. Additionally, the pattern of activity in these brain regions (bilateral TPJ, DMPFC and bilateral STS) did not change after controlling for variations in population density; in fact, the contact effects tended to get slightly stronger after controlling for this factor (see Supplementary Discussion 4). Although a significant four-way interaction effect (task × target race × lifetime contact × population density) in bilateral amygdalae was also obtained, due to sample size limitations we restrict our interpretation of this result. Notably, we again failed to find any evidence that differences in contact led to differential brain activity in brain regions supporting social cognition for other-race faces.

Task PLS analysis revealed two distinct networks associated with either the social task or the non-social task. During the social task, participants showed greater co-activation in a network of regions associated with social cognition including TPJ and both dorsal and ventral aspects of MPFC, whereas during the non-social task participants showed greater co-activation in a network of regions involved in visual processing, including extensive occipital regions and fusiform gyrus. These results support the validity of the task utilized to contrast social and non-social processes. Indeed, the social task recruited brain regions previously shown to be involved in social cognition, and the non-social task recruited brain regions involved in perceptual face processing and attention.

We also used network analysis to explore the relationship between brain network activation to each condition and the interracial contact of perceivers. This analysis revealed complementary patterns of results. The first network identified (i.e. first LV) was driven by changes in recruitment of brain regions during non-social trials associated with lifetime contact. When rating the symmetry of either Black or White faces, an ostensibly novel task for perceivers, increased contact was associated with greater co-activation of networks of brain regions involved in face processing and salience detection, e.g. bilateral insula and ACC (Seeley *et al.*, 2007; Menon and Uddin, 2010; Uddin, 2015). Explicitly rating the symmetry of others’ faces preferentially relies on featural processing whereas making a social inference from faces is a more familiar task that may not rely on featural processing. Increased co-activation in brain regions sensitive to face processing and salience detection during the novel symmetry-rating task may in part reflect high-contact perceivers’ tendency to rely less on featural processing than low-contact perceivers (Hancock and Rhodes, 2008; Walker *et al.*, 2008; Rhodes *et al.*, 2009). Accordingly, these findings suggest that the performance of a novel face symmetry task may be relatively more salient to high-contact perceivers.

The second pattern (i.e. second LV) was driven by changes in recruitment of brain regions during all experimental conditions associated with lifetime contact. Two distinct networks contributed to this pattern. The first, more extensive network showed decreased co-activation with increasing contact. Similar to the results obtained by Cloutier *et al.* (2017), we found that higher levels of contact were associated with decreased overall recruitment of a network of brain regions involved in face processing (e.g. fusiform gyrus) and social cognition (e.g. MPFC). The second, less extensive network showed increased co-activation with increasing contact. This network involved only the superior occipital gyrus and brainstem nuclei. We again found no evidence for race differences in face processing as a function of contact.

Consistent across all analyses and with previous work examining the impact of contact on the neural substrates of face perception (i.e. Cloutier *et al.*, 2017), we fail to find evidence that brain regions supporting social cognition are differentially recruited as a function of race during social inferences from faces. Although there is strong evidence that contact shapes peoples’ evaluative responses toward outgroup members (e.g. Pettigrew and Tropp, 2006; Tausch and Hewstone, 2010; Pettigrew *et al.*, 2011), high-contact perceivers do not appear to process outgroup faces differently than ingroup faces during a social task. Rather, they process ‘all’ faces differently than low-contact perceivers; more specifically, these high-contact perceivers seem to be more cognitively efficient face processors who experience all faces, regardless of race, as less socially salient stimuli. These results suggest that perceivers differentially process race as a function of their experience with a variety of faces in general, at least in the context in which these faces are encountered.

It is also important to note that the present study used an exclusively U.S.-based White non-Hispanic sample. By focusing on the impact of contact on White non-Hispanic group members, we limit our ability to generalize these results to a broader, more diverse population. Although we would expect similar increases in interracial contact to have the same impact on neural responses during social judgments from faces for all perceivers, determining whether these effects replicate in samples that include non-White and/or Hispanic group members is a critical next step for future research.

Overall, these results are consistent with the possibility that interracial contact broadly influences the neural substrates involved in social cognition. In addition to its well-characterized effects on reducing evaluative biases toward outgroup members, contact may importantly further shape basic social cognitive processes because of changes in face processing efficiency and salience detection. Contact may therefore influence a broader array of social cognitive processes than initially thought.

## Supplementary Material

nsab090_Supp

## Data Availability

*Data are available on request from the corresponding author without conditions.*

## Footnotes

1 The term ‘people’ was replaced depending on the age range for that section of the contact questionnaire. For the 0–6-year-old and 7–12-year-old age ranges, ‘people’ was replaced with ‘children’. For the 13–18-year-old age range, ‘people’ was replaced with ‘teens’. For the current age range, ‘people’ was replaced with ‘peers’.

2 We note that testing simple slopes using ±1 s.d. is traditional as per guidelines proposed by Cohen and colleagues (Cohen *et al.*, 1983). However, these guidelines were suggested for use in the context of regression models in which no a priori meaningful values exist (West *et al.*, 1996). Based on previous piloting in our lab, we selected ±2 s.d. values as meaningful points for comparison for the present study.

3 We provide the statistics for these non-significant slopes in this footnote. Right TPJ non-social: *B *= 0.177, SE* *= 0.106, *df *= 74.420, *t*-value = 1.665, *P** *= 0.100, 95% CI = [−0.031, 0.385]; social: *B *= −0.038, SE* *= 0.106, *df *= 74.420, *t*-value = −0.355, *P** *= 0.723, 95% CI = [−0.246, 0.170]. DMPFC non-social: *B *= 0.331, SE* *= 0.184, *df* = 76.220, *t*-value = 1.793, *P* = 0.077, 95% CI = [−0.031, 0.692]; social: *B *= −0.174, SE* *= 0.184, *df* = 76.220, *t*-value = −0.943, *P** *= 0.349, 95% CI = [−0.535, 0.188]. Left amygdala non-social: *B *= 0.220, SE* *= 0.123, *df *= 80.440, *t*-value = 1.790, *P** *= 0.077, 95% CI = [−0.021, 0.461]; social: *B *= −0.166, SE* *= 0.123, *df *= 80.440, *t*-value = −1.354, *P** *= 0.179, 95% CI = [−0.407, 0.074].
