# Supplementary material for: Interracial contact differentially shapes brain networks involved in social and non-social judgments from faces: a combination of univariate and multivariate approaches
Source: Soc Cogn Affect Neurosci. 2021 Jul 28;17(2):218–30. doi: 10.1093/scan/nsab090 (PMC8847903; doi:10.1093/scan/nsab090)
Supplement: nsab090_Supp [file nsab090_supp.zip › scan-21-017-File009.docx]

**SUPPLEMENTARY INFORMATION SECTIONS 1–6**

Interracial contact differentially shapes brain networks involved in social and non-social judgments from faces:

A combination of univariate and multivariate approaches.

**Supplementary Discussion 1. Social-Cognitive Measures for Unrelated Resting-State and Anatomical Analyses.**

In addition to the childhood and current interracial contact questionnaire, we also assessed a number of more general social-cognitive measures for analyses of a large-scale resting state and structural imaging dataset. These measures were collected prior to the scanning session for this study. We report a complete list of these measures here for interested readers.

- Altruism scale (Rushton et al., 1981)
- Behavioral Inhibition System/Behavioral Activation System (BIS/BAS) scale (Carver & White, 1994)
- Cambridge Face Memory Test (Duchaine & Nakayama, 2006)
- Connor-Davidson Resilience Scale (CD-RISC: Connor & Davidson, 2003)
- Heider & Simmel mentalizing animations (Heider & Simmel, 1944)
- Geneva Emotion Recognition Test (GERT-S: Schlegel & Scherer, 2016)
- Mind wandering and fidgeting scales (Carriere et al., 2013)
- Questionnaire of Cognitive and Affective Empathy (QCAE: Reniers, Corcoran, Drake, Shryane, & Völlm, 2011)
- Revised UCLA Loneliness Scale (R-UCLA: Russell, Peplau, & Cutrona, 1980)
- Risk Taking Index (RTI: Nicholson, Soane, Fenton‐O’Creevy, & Willman, 2005)
- Sense of power scale (Anderson et al., 2011)
- Social comparison scale (Allan & Gilbert, 1995)
- Trust in people scale (Yamagishi & Yamagishi, 1994)
- Modern Racism Scale (McConahay, 1986)
- Race Implicit Association Test (Greenwald et al., 1998, 2003)
- External/Internal Motivation to Respond without Prejudice (Plant & Devine, 1998)

**Supplementary Discussion 2. Stimulus Presentation Optimization.**

This fMRI protocol used a blocked rapid event-related design; within each interest or symmetry rating block, Black and White trials were presented in a rapid event-related manner. We used optseq2 to optimize the intertrial intervals (varied from 0 to 6 seconds) within each block. The following code was used for each block:

./optseq2 --ntp 60 --tr 2 --psdwin 0 20 --ev evt1 6 8 --ev evt2 6 7 --o intsym --nkeep 3 --nsearch 10000 --tnullmin 0 --tnullmax 6

./optseq2 --ntp 60 --tr 2 --psdwin 0 20 --ev evt1 6 7 --ev evt2 6 8 --o intsym --nkeep 3 --nsearch 10000 --tnullmin 0 --tnullmax 6

Each event was 6 seconds to allow for 4 seconds of stimulus presentation and 2 seconds of response window. The order of the four runs was randomized for each participant. We selected the first possible jittering sequence that did not include more than three consecutive trials of either race.

**Supplementary Discussion 3. ROI Analyses for Childhood and Current Contact Separately**

Because childhood and current contact were only moderately correlated, here we report all confirmatory ROI analyses for childhood and current contact difference scores separately. Overall results are similar to those for the combined lifetime contact measure reported in the main text with some minor differences.

**Analyses Using Childhood Contact as a Predictor**

Regression results predicting BOLD activity in all *a priori* ROIs using target race, task, childhood contact, and all possible interactions as predictors are presented in Supplementary Table 1. In general, most results found using lifetime contact as a predictor remained when using childhood contact as a predictor. However, the task x contact interaction was no longer significant in right TPJ. The task x contact interaction remained significant in DMPFC and left amygdala.

Low (*B* = 1.505, *SE* = 0.351, *t*(156) = 4.286, *p* < 0.001, 95% CI = [0.817, 2.194]) and average (*B* = 0.546, *SE* = 0.157, *t*(156) = 3.484, *p* < 0.001, 95% CI = 0.239, 0.854]) contact perceivers recruited DMPFC significantly more during social trials than non-social trials, whereas high (*B* = -0.413, *SE* = 0.351, *t*(156) = -1.175, *p* = 0.242, 95% CI = [-1.101, 0.276]) contact perceivers recruited DMPFC equally regardless of task (see Supplementary Figure 1A). The slope was significant during social trials (*B* = -0.403, *SE* = 0.183, *t*(76.716) = -2.195, *p* = 0.031, 95% CI = [-0.762, -0.043]), but not non-social trials (*B* = 0.077, *SE* = 0.183, *t*(76.716) = 0.420, *p* = 0.676, 95% CI = [-0.282, 0.437]; see Supplementary Figure 1A).

Low (*B* = 1.118, *SE* = 0.246, *t*(156) = 4.545, *p* < 0.001, 95% CI = [0.636, 1.601]) and average (*B* = 0.302, *SE* = 0.110, *t*(156) = 2.758, *p* = 0.001, 95% CI = [0.087, 0.517]) contact perceivers recruited left amygdala significantly more during social trials than non-social trials, whereas high (*B* = -0.515, *SE* = 0.246, *t*(156) = -2.091, *p* = 0.038, 95% CI = [-0.997, -0.032]) contact perceivers recruited left amygdala significantly more during non-social trials than social trials (see Supplementary Figure 1B). The slopes in both the social (*B* = -0.199, *SE* = 0.123, *t*(79.844) = -1.619, *p* = 0.109, 95% CI = [-0.439, 0.042]) and non-social (*B* = 0.210, *SE* = 0.123, *t*(79.844) = 1.709, *p* = 0.091, 95% CI = [-0.031, 0.450]) conditions were not significant (see Supplementary Figure 1B).

**Supplementary Table 1.** ROI analysis results using the *z*-scored childhood contact difference score as a predictor. L = left and R = right. Significant results are marked with an asterisk, *p* < 0.05.

| **Predictors** | ***B*** | ***SE*** | **df** | **95% CI** | ***t-*value** | ***p*-value** |
| --- | --- | --- | --- | --- | --- | --- |
| *L. TPJ* | | | | | | |
| (Intercept) | -1.095 | 0.115 | 52 | [-1.320, -0.869] | -9.518 | < 0.001 * |
| Target race | 0.046 | 0.102 | 156 | [-0.154, 0.246] | 0.447 | 0.655 |
| Task | 0.641 | 0.102 | 156 | [0.441, 0.842] | 6.284 | < 0.001 * |
| Childhood contact | 0.167 | 0.115 | 52 | [-0.059, 0.393] | 1.452 | 0.153 |
| Target race x task | -0.107 | 0.204 | 156 | [-0.507, 0.293] | -0.522 | 0.602 |
| Target race x childhood contact | -0.047 | 0.102 | 156 | [-0.247, 0.154] | -0.458 | 0.648 |
| Task x childhood contact | -0.178 | 0.102 | 156 | [-0.379, 0.022] | -1.744 | 0.083 |
| Target race x task x childhood contact | 0.018 | 0.205 | 156 | [-0.383, 0.419] | 0.087 | 0.931 |
| *R. TPJ* |  |  |  |  |  |  |
| (Intercept) | -0.848 | 0.096 | 52 | [-1.037, -0.660] | -8.818 | < 0.001 * |
| Target race | -0.030 | 0.089 | 156 | [-0.203, 0.144] | -0.333 | 0.739 |
| Task | 0.778 | 0.089 | 156 | [0.604, 0.952] | 8.772 | < 0.001 * |
| Childhood contact | 0.091 | 0.096 | 52 | [-0.098, 0.280] | 0.945 | 0.349 |
| Target race x task | -0.004 | 0.177 | 156 | [-0.352, 0.344] | -0.024 | 0.981 |
| Target race x childhood contact | -0.051 | 0.089 | 156 | [-0.225, 0.123] | -0.573 | 0.567 |
| Task x childhood contact | -0.086 | 0.089 | 156 | [-0.260, 0.089] | -0.964 | 0.336 |
| Target race x task x childhood contact | 0.007 | 0.178 | 156 | [-0.342, 0.355] | 0.038 | 0.970 |
| *DMPFC* |  |  |  |  |  |  |
| (Intercept) | -0.653 | 0.165 | 52 | [-0.977, -0.329] | -3.949 | < 0.001 * |
| Target race | -0.326 | 0.157 | 156 | [-0.634, -0.019] | -2.080 | 0.039 * |
| Task | 0.546 | 0.157 | 156 | [0.239, 0.854] | 3.484 | 0.001 * |
| Childhood contact | -0.163 | 0.166 | 52 | [-0.488, 0.162] | -0.982 | 0.331 |
| Target race x task | -0.281 | 0.314 | 156 | [-0.895, 0.334] | -0.895 | 0.372 |
| Target race x childhood contact | -0.030 | 0.157 | 156 | [-0.338, 0.278] | -0.190 | 0.850 |
| Task x childhood contact | -0.480 | 0.157 | 156 | [-0.788, -0.172] | -3.052 | 0.003 * |
| Target race x task x childhood contact | -0.140 | 0.314 | 156 | [-0.756, 0.476] | -0.444 | 0.657 |
| *L. STS* |  |  |  |  |  |  |
| (Intercept) | -0.913 | 0.092 | 52 | [-1.094, -0.733] | -9.913 | < 0.001 * |
| Target race | -0.072 | 0.099 | 156 | [-0.266, 0.121] | -0.734 | 0.464 |
| Task | 0.423 | 0.099 | 156 | [0.229, 0.617] | 4.281 | < 0.001 * |
| Childhood contact | -0.085 | 0.092 | 52 | [-0.266, 0.096] | -0.921 | 0.362 |
| Target race x task | -0.350 | 0.198 | 156 | [-0.737, 0.037] | -1.773 | 0.078 |
| Target race x childhood contact | -0.054 | 0.099 | 156 | [-0.248, 0.140] | -0.544 | 0.587 |
| Task x childhood contact | -0.194 | 0.099 | 156 | [-0.388, 0.000] | -1.961 | 0.052 |
| Target race x task x childhood contact | 0.027 | 0.198 | 156 | [-0.361, 0.416] | 0.138 | 0.890 |
| *R. STS* |  |  |  |  |  |  |
| (Intercept) | -1.060 | 0.088 | 52 | [-1.233, -0.886] | -11.980 | < 0.001 * |
| Target race | 0.005 | 0.105 | 156 | [-0.202, 0.211] | 0.043 | 0.966 |
| Task | 0.659 | 0.105 | 156 | [0.452, 0.865] | 6.252 | < 0.001 * |
| Childhood contact | 0.052 | 0.089 | 52 | [-0.122, 0.226] | 0.589 | 0.558 |
| Target race x task | -0.187 | 0.211 | 156 | [-0.600, 0.226] | -0.888 | 0.376 |
| Target race x childhood contact | -0.105 | 0.106 | 156 | [-0.312, 0.102] | -0.992 | 0.323 |
| Task x childhood contact | -0.172 | 0.106 | 156 | [-0.379, 0.035] | -1.633 | 0.105 |
| Target race x task x childhood contact | -0.082 | 0.211 | 156 | [-0.496, 0.332] | -0.388 | 0.699 |
| *L. amygdala* |  |  |  |  |  |  |
| (Intercept) | 0.074 | 0.109 | 52 | [-0.140, 0.289] | 0.678 | 0.501 |
| Target race | 0.017 | 0.110 | 156 | [-0.199, 0.232] | 0.153 | 0.879 |
| Task | 0.302 | 0.110 | 156 | [0.087, 0.517] | 2.748 | 0.007 * |
| Childhood contact | 0.006 | 0.110 | 52 | [-0.209, 0.220] | 0.051 | 0.960 |
| Target race x task | 0.128 | 0.220 | 156 | [-0.303, 0.558] | 0.581 | 0.562 |
| Target race x childhood contact | -0.100 | 0.110 | 156 | [-0.316, 0.116] | -0.910 | 0.364 |
| Task x childhood contact | -0.408 | 0.110 | 156 | [-0.624, -0.192] | -3.708 | < 0.001 * |
| Target race x task x childhood contact | 0.254 | 0.220 | 156 | [-0.178, 0.686] | 1.153 | 0.251 |
| *R. amygdala* |  |  |  |  |  |  |
| (Intercept) | 0.522 | 0.125 | 52 | [0.278, 0.767] | 4.180 | < 0.001 * |
| Target race | 0.028 | 0.123 | 156 | [-0.213, 0.269] | 0.227 | 0.821 |
| Task | 0.257 | 0.123 | 156 | [0.016, 0.498] | 2.087 | 0.038 * |
| Childhood contact | 0.175 | 0.125 | 52 | [-0.070, 0.421] | 1.399 | 0.168 |
| Target race x task | 0.134 | 0.246 | 156 | [-0.349, 0.616] | 0.543 | 0.588 |
| Target race x childhood contact | -0.154 | 0.123 | 156 | [-0.396, 0.088] | -1.247 | 0.214 |
| Task x childhood contact | -0.162 | 0.123 | 156 | [-0.404, 0.080] | -1.315 | 0.190 |
| Target race x task x childhood contact | 0.165 | 0.247 | 156 | [-0.319, 0.648] | 0.667 | 0.506 |

A.
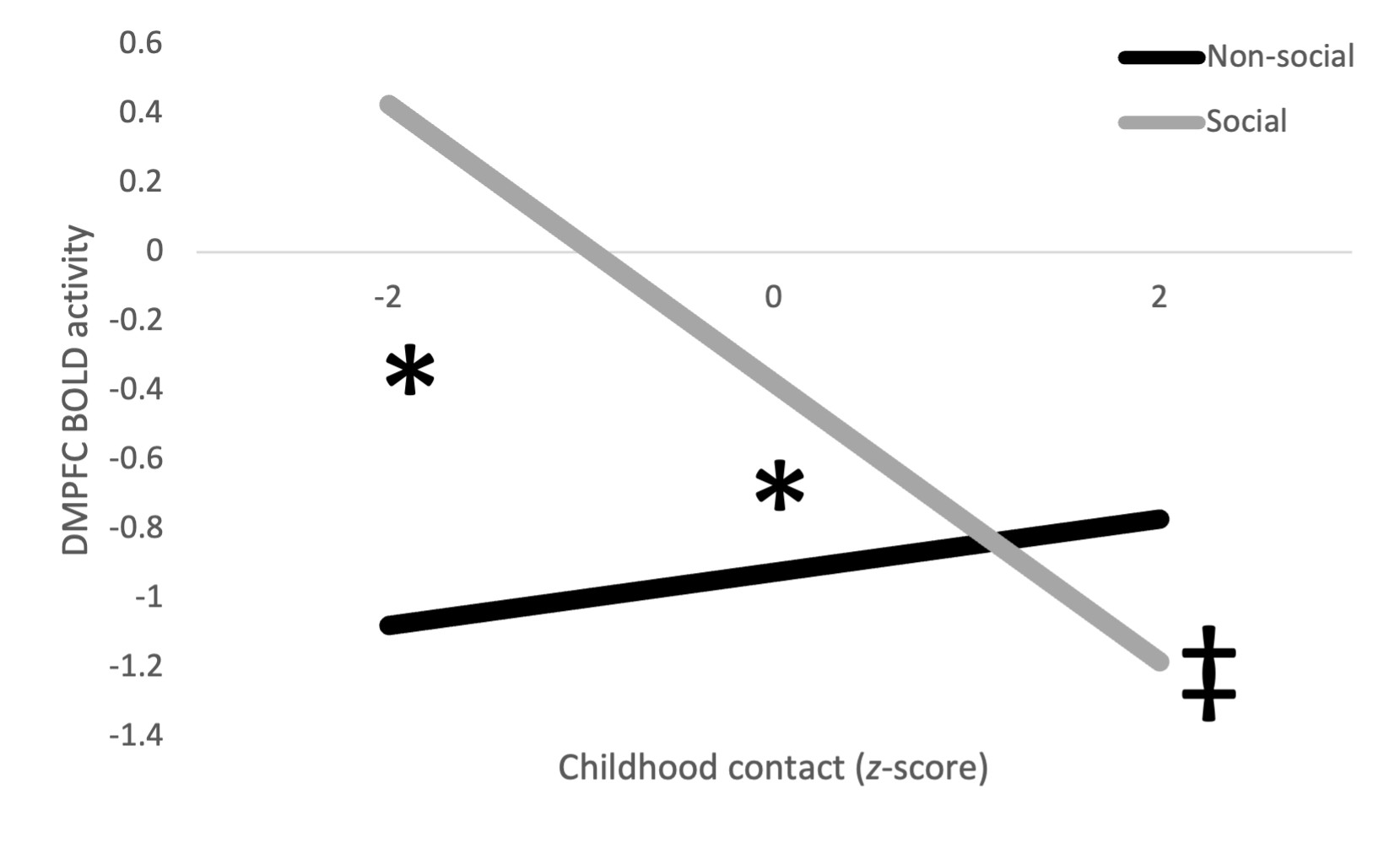
 B. **
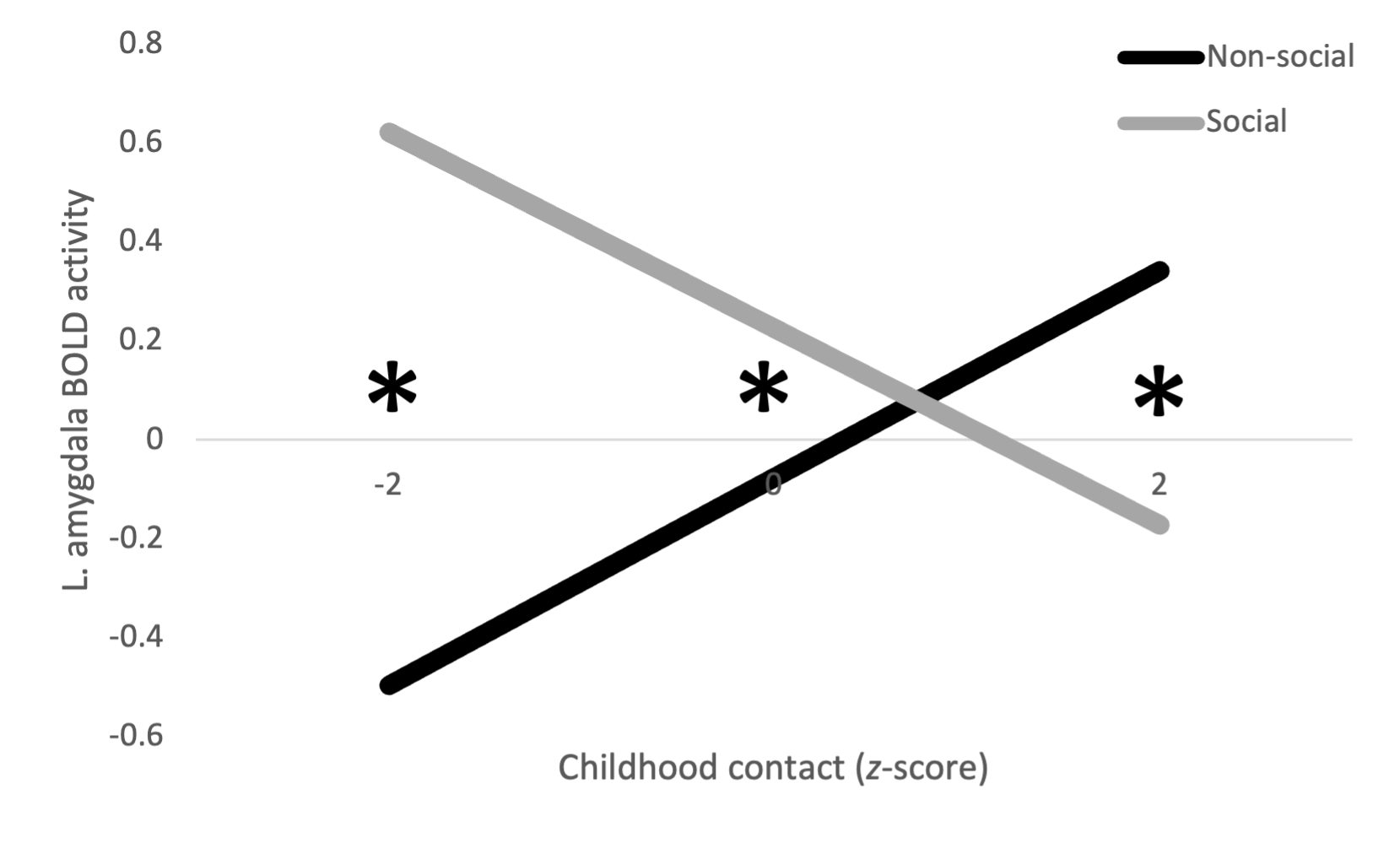
**

**Supplementary Figure 1.**  The two-way interaction of task and childhood contact significantly predicted BOLD activity in (a) DMPFC and (b) left amygdala. Significant simple differences are marked by an asterisk (*). Significant slopes are marked by a double cross (‡).

**Analyses Using Current Contact as a Predictor**

Regression results predicting BOLD activity in all *a priori* ROIs using target race, task, current contact, and all possible interactions as predictors are presented in Supplementary Table 2. As seen with childhood contact, most results found using lifetime contact as a predictor remained when using current contact as a predictor. Notably, the task x contact interaction remained significant in right TPJ, DMPFC and left amygdala (see Supplementary Figure 2A, B, and C). We also observed a significant main effect of current contact in the right amygdala; this effect did not emerge when using lifetime or childhood contact as predictors.

In right TPJ, low (*B* = 1.245, *SE* = 0.195, *t*(156) = 6.386, *p* < 0.001, 95% CI = [0.683, 1.627]) and average (*B* = 0.778, *SE* = 0.087, *t*(156) = 8.944, *p* < 0.001, 95% CI = [0.608, 0.949]) contact perceivers recruited this region significantly more during social than non-social trials; high (*B* = 0.312, *SE* = 0.195, *t*(156) = 1.599, *p* = 0.112, 95% CI = [-0.070, 0.694]) contact perceivers did not significantly differ regardless of task (see Supplementary Figure 2A). Slopes in the social (*B* = -0.081, *SE* = 0.106, *t*(74.070) = -0.758, *p* = 0.451, 95% CI = [-0.289, 0.128]) and non-social (*B* = 0.153, *SE* = 0.106, *t*(74.070) = 1.433, *p* = 0.156, 95% CI = [-0.056, 0.361]) condition were not significant (see Supplementary Figure 2A).

In DMPFC, low (*B* = 1.295, *SE* = 0.356, *t*(156) = 3.641, *p* < 0.001, 95% CI = [0.598, 1.992]) and average (*B* = 0.546, *SE* = 0.159, *t*(145) = 3.442, *p* < 0.001, 95% CI = [0.235, 0.857]) contact perceivers recruited this region significantly more during social than non-social trials; high (*B* = -0.202, *SE* = 0.356, *t*(156) = -0.569, *p* = 0.571, 95% CI = [-0.899, 0.495]) contact perceivers did not significantly differ regardless of task (see Supplementary Figure 2B). The slope during social (*B* = 0.020, *SE* = 0.183, *t*(77.652) = 0.107, *p* = 0.915, 95% CI = [-0.339, 0.378]) trials was not significant; however, during non-social (*B* = 0.394, *SE* = 0.183, *t*(77.652) = 2.152, *p* = 0.034, 95% CI = [0.035, 0.752]) trials there was a significant positive slope (see Supplementary Figure 2B).

In left amygdala, low (*B* = 0.824, *SE* = 0.254, *t*(156) = 3.245, *p* = 0.001, 95% CI = [0.326, 1.321]) and average (*B* = 0.302, *SE* = 0.113, *t*(156) = 2.664, *p* < 0.001, 95% CI = [0.080, 0.524]) contact perceivers recruited this region significantly more during social than non-social trials; high (*B* = -0.220, *SE* = 0.254, *t*(156) = -0.866, *p* = 0.388, 95% CI = [-0.717, 0.278]) contact perceivers did not significantly differ regardless of task (see Supplementary Figure 2C). Slopes in the social (*B* = -0.098, *SE* = 0.123, *t*(81.747) = -0.795, *p* = 0.429, 95% CI = [-0.340, 0.144]) and non-social (*B* = 0.163, *SE* = 0.123, *t*(81.747) = 1.319, *p* = 0.191, 95% CI = [-0.079, 0.405]) condition were not significant (see Supplementary Figure 2C).

**Supplementary Table 2.** ROI analysis results using the *z*-scored current contact difference score as a predictor. L = left and R = right. Significant results are marked with an asterisk, *p* < 0.05.

| **Predictors** | ***B*** | ***SE*** | **df** | **95% CI** | ***t-*value** | ***p*-value** |
| --- | --- | --- | --- | --- | --- | --- |
| *L. TPJ* | | | | | | |
| (Intercept) | -1.095 | 0.117 | 52 | [-1.325, -0.865] | -9.336 | < 0.001 * |
| Target race | 0.046 | 0.103 | 156 | [-0.156, 0.248] | 0.443 | 0.659 |
| Task | 0.641 | 0.103 | 156 | [0.439, 0.844] | 6.222 | < 0.001 * |
| Current contact | -0.028 | 0.118 | 52 | [-0.259, 0.202] | -0.242 | 0.810 |
| Target race x task | -0.107 | 0.206 | 156 | [-0.511, 0.297] | -0.517 | 0.606 |
| Target race x current contact | 0.017 | 0.103 | 156 | [-0.185, 0.220] | 0.166 | 0.869 |
| Task x current contact | -0.027 | 0.103 | 156 | [-0.230, 0.175] | -0.264 | 0.792 |
| Target race x task x current contact | 0.049 | 0.207 | 156 | [-0.356, 0.454] | 0.235 | 0.814 |
| *R. TPJ* |  |  |  |  |  |  |
| (Intercept) | -0.848 | 0.097 | 52 | [-1.038, -0.658] | -8.754 | < 0.001 * |
| Target race | -0.030 | 0.087 | 156 | [-0.200, 0.141] | -0.340 | 0.734 |
| Task | 0.778 | 0.087 | 156 | [0.608, 0.949] | 8.944 | < 0.001 * |
| Current contact | 0.036 | 0.097 | 52 | [-0.154, 0.226] | 0.370 | 0.713 |
| Target race x task | -0.004 | 0.174 | 156 | [-0.345, 0.337] | -0.024 | 0.981 |
| Target race x current contact | 0.018 | 0.087 | 156 | [-0.153, 0.189] | 0.208 | 0.835 |
| Task x current contact | -0.233 | 0.087 | 156 | [-0.494, -0.062] | -2.675 | 0.008 * |
| Target race x task x current contact | 0.094 | 0.174 | 156 | [-0.248, 0.436] | 0.539 | 0.591 |
| *DMPFC* |  |  |  |  |  |  |
| (Intercept) | -0.653 | 0.164 | 52 | [-0.975, -0.331] | -3.971 | < 0.001 * |
| Target race | -0.326 | 0.159 | 156 | [-0.637, -0.015] | -2.055 | 0.042 * |
| Task | 0.546 | 0.159 | 156 | [0.235, 0.857] | 3.442 | 0.001 * |
| Current contact | 0.207 | 0.165 | 52 | [-0.116, 0.530] | 1.254 | 0.215 |
| Target race x task | -0.281 | 0.317 | 156 | [-0.903, 0.342] | -0.884 | 0.378 |
| Target race x current contact | -0.015 | 0.159 | 156 | [-0.327, 0.297] | -0.096 | 0.924 |
| Task x current contact | -0.374 | 0.159 | 156 | [-0.686, -0.062] | -2.352 | 0.020 * |
| Target race x task x current contact | -0.031 | 0.318 | 156 | [-0.654, 0.593] | -0.096 | 0.923 |
| *L. STS* |  |  |  |  |  |  |
| (Intercept) | -0.913 | 0.092 | 52 | [-1.093, -0.733] | -9.960 | < 0.001 * |
| Target race | -0.072 | 0.100 | 156 | [-0.268, 0.123] | -0.725 | 0.469 |
| Task | 0.423 | 0.100 | 156 | [0.227, 0.619] | 4.233 | < 0.001 * |
| Current contact | -0.107 | 0.092 | 52 | [-0.287, 0.073] | -1.163 | 0.250 |
| Target race x task | -0.350 | 0.200 | 156 | [-0.742, 0.041] | -1.753 | 0.082 |
| Target race x current contact | 0.039 | 0.100 | 156 | [-0.157, 0.236] | 0.393 | 0.695 |
| Task x current contact | -0.055 | 0.100 | 156 | [-0.251, 0.142] | -0.546 | 0.586 |
| Target race x task x current contact | -0.070 | 0.200 | 156 | [-0.462, 0.323] | -0.348 | 0.729 |
| *R. STS* |  |  |  |  |  |  |
| (Intercept) | -1.060 | 0.088 | 52 | [-1.232, -0.888] | -12.072 | < 0.001 * |
| Target race | 0.005 | 0.106 | 156 | [-0.203, 0.212] | 0.043 | 0.966 |
| Task | 0.659 | 0.106 | 156 | [0.451, 0.866] | 6.231 | < 0.001 * |
| Current contact | 0.095 | 0.088 | 52 | [-0.078, 0.267] | 1.074 | 0.288 |
| Target race x task | -0.187 | 0.211 | 156 | [-0.601, 0.227] | -0.885 | 0.378 |
| Target race x current contact | 0.007 | 0.106 | 156 | [-0.201, 0.214] | 0.064 | 0.949 |
| Task x current contact | -0.146 | 0.106 | 156 | [-0.354, 0.061] | -1.380 | 0.170 |
| Target race x task x current contact | -0.192 | 0.212 | 156 | [-0.607, 0.223] | -0.907 | 0.366 |
| *L. amygdala* |  |  |  |  |  |  |
| (Intercept) | 0.074 | 0.109 | 52 | [-0.140, 0.288] | 0.679 | 0.500 |
| Target race | 0.017 | 0.113 | 156 | [-0.205, 0.239] | 0.148 | 0.882 |
| Task | 0.302 | 0.113 | 156 | [0.080, 0.524] | 2.664 | 0.009 * |
| Current contact | 0.032 | 0.110 | 52 | [-0.182, 0.247] | 0.295 | 0.769 |
| Target race x task | 0.128 | 0.227 | 156 | [-0.317, 0.572] | 0.563 | 0.574 |
| Target race x current contact | 0.050 | 0.114 | 156 | [-0.173, 0.273] | 0.441 | 0.660 |
| Task x current contact | -0.261 | 0.114 | 156 | [-0.484, -0.038] | -2.297 | 0.023 * |
| Target race x task x current contact | 0.058 | 0.227 | 156 | [-0.387, 0.503] | 0.254 | 0.800 |
| *R. amygdala* |  |  |  |  |  |  |
| (Intercept) | 0.522 | 0.122 | 52 | [0.283, 0.762] | 4.275 | < 0.001 * |
| Target race | 0.028 | 0.124 | 156 | [-0.216, 0.272] | 0.224 | 0.823 |
| Task | 0.257 | 0.124 | 156 | [0.013, 0.501] | 2.065 | 0.041 * |
| Current contact | -0.258 | 0.122 | 52 | [-0.498, -0.018] | -2.106 | 0.040 * |
| Target race x task | 0.134 | 0.249 | 156 | [-0.354, 0.621] | 0.537 | 0.592 |
| Target race x current contact | -0.066 | 0.125 | 156 | [-0.310, 0.178] | -0.529 | 0.597 |
| Task x current contact | -0.011 | 0.125 | 156 | [-0.255, 0.233] | -0.087 | 0.931 |
| Target race x task x current contact | -0.066 | 0.249 | 156 | [-0.555, 0.422] | -0.266 | 0.790 |

A.
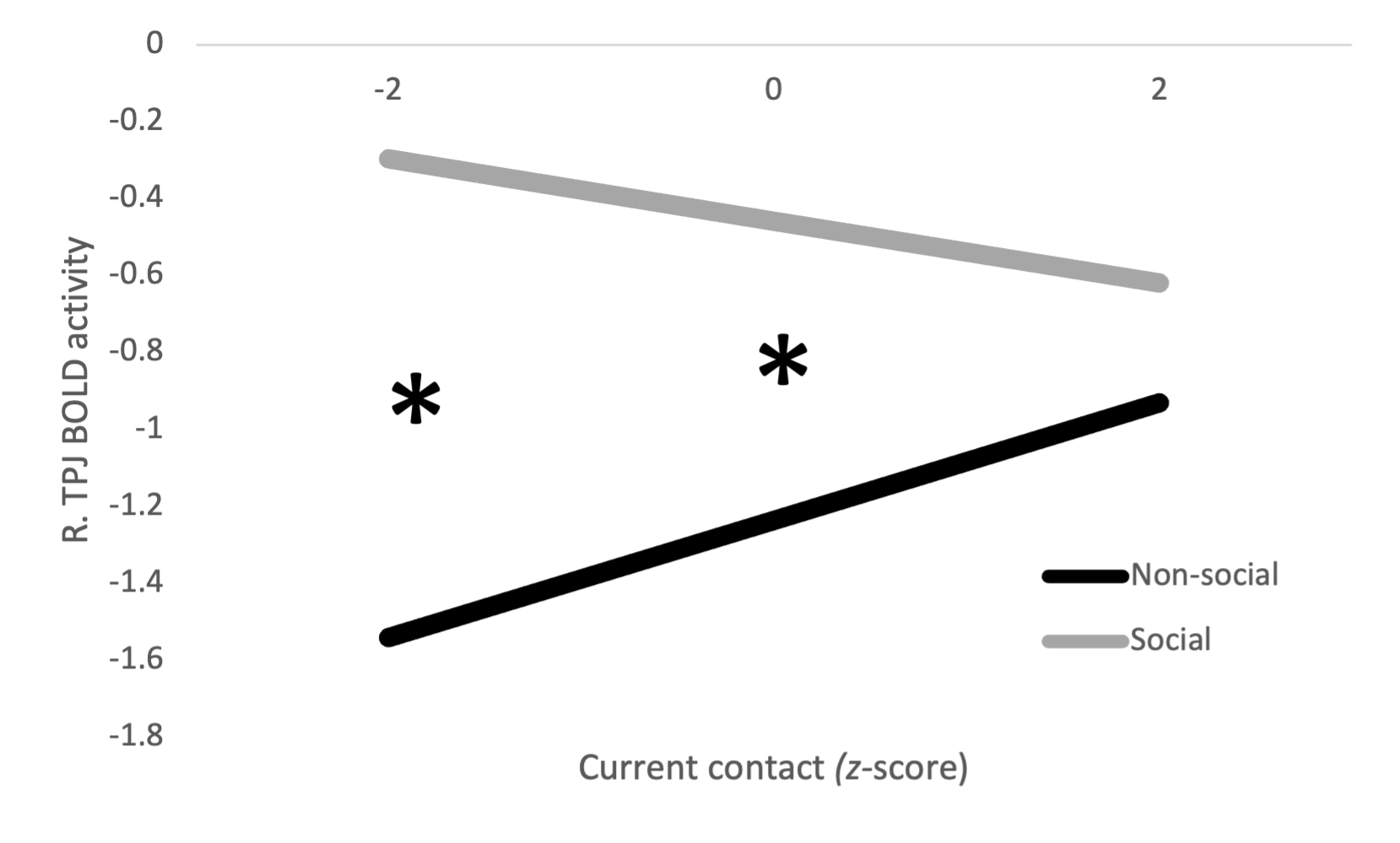
 B.
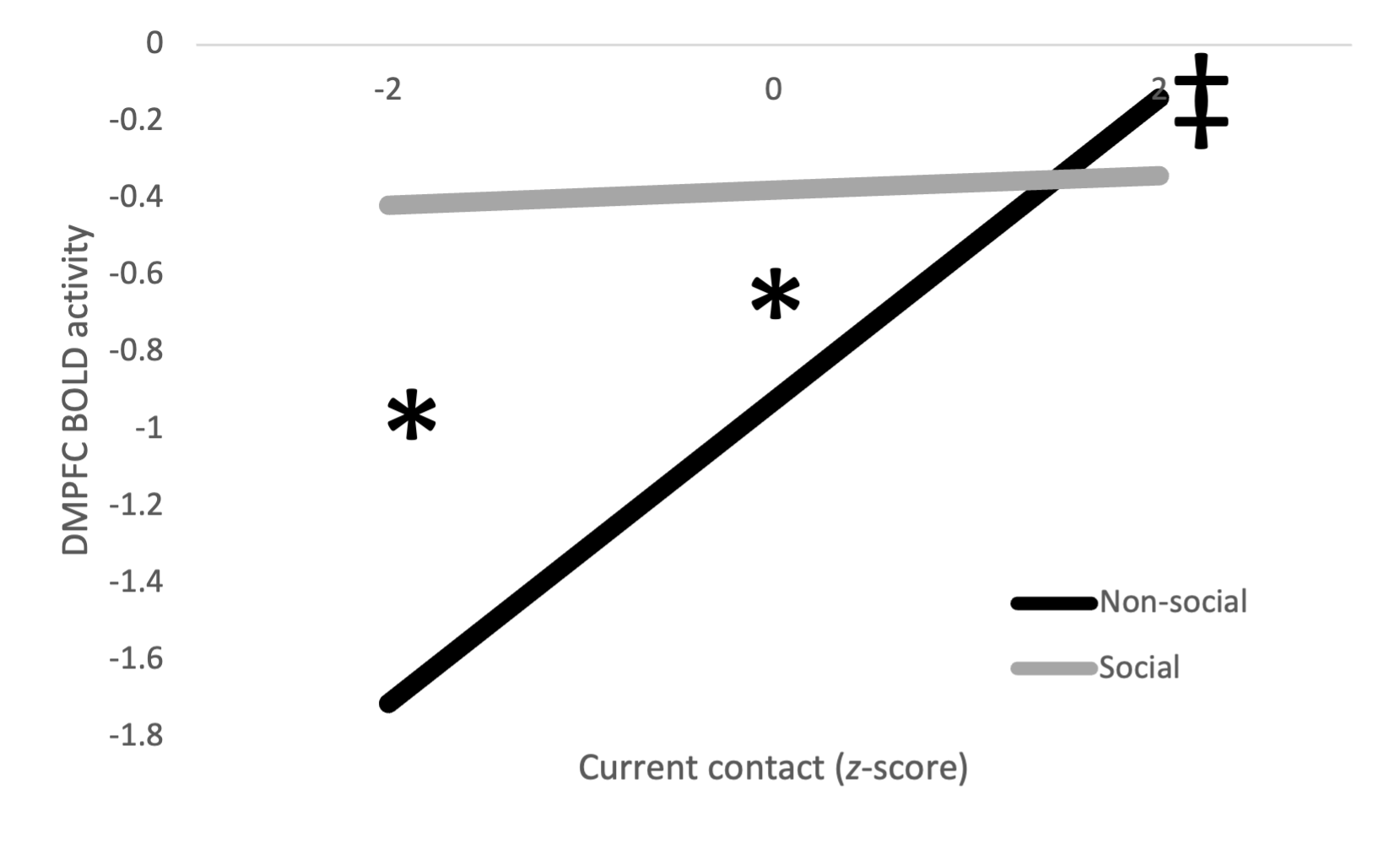


C.
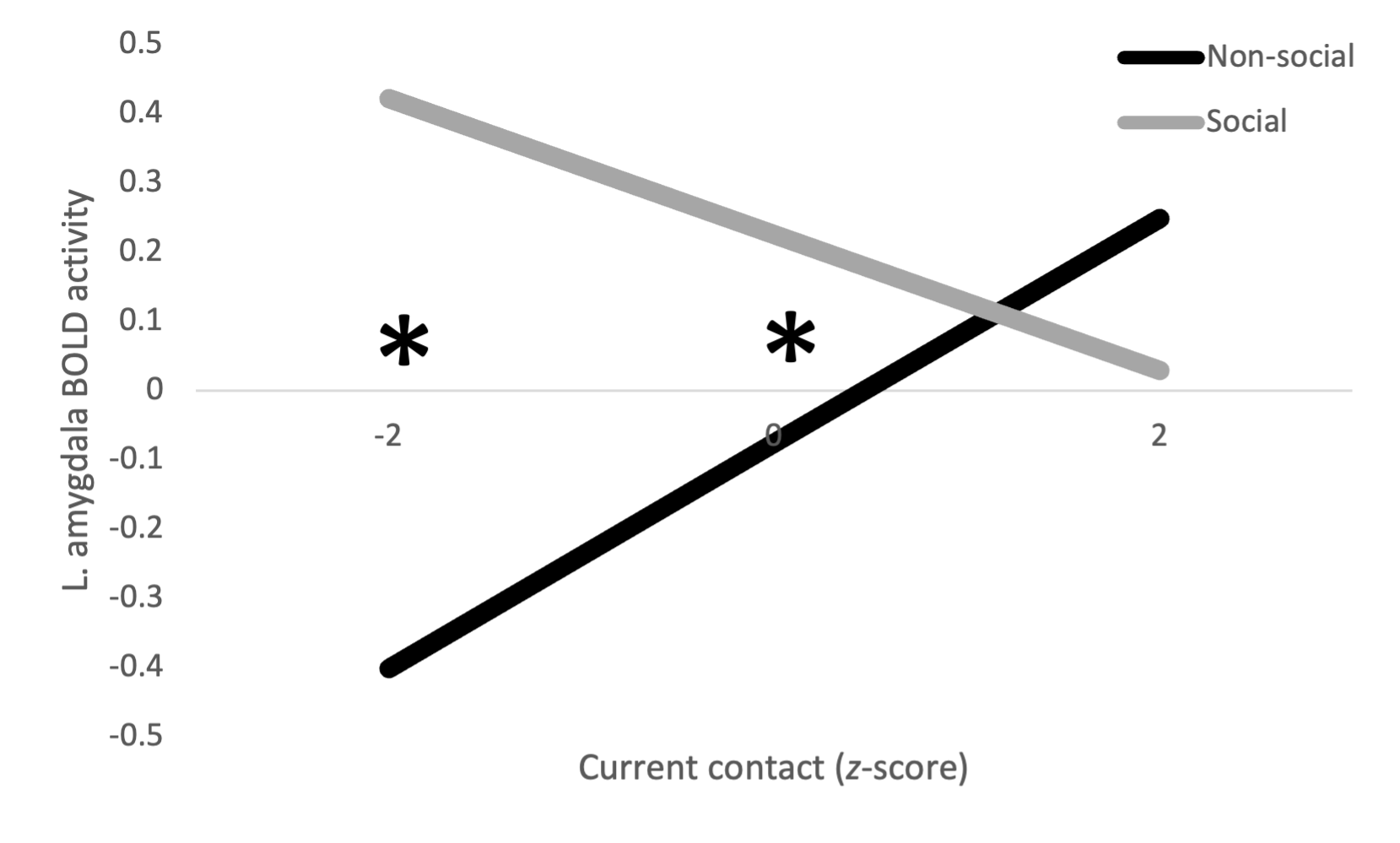


**Supplementary Figure 2.**  The two-way interaction of task and childhood contact significantly predicted BOLD activity in (a) right TPJ, (b) DMPFC, and (c) left amygdala. Significant simple differences are marked by an asterisk (*). Significant slopes are marked by a double cross (‡).

**Supplementary Discussion 4. Population Density Analyses**

To account for the possibility that any potential effects of contact may be driven by exposure to more faces in general rather than other-race contact specifically, we used participants’ zip codes to infer the population density of the areas where they resided throughout their lives (0-6 years old, 7-12 years old, 13-18 years old, and currently). To infer population density during childhood, we used participants’ self-reported zip codes cross-referenced against their city/town and state name to confirm accurate zip code recall. To infer current population density, we used the latitude and longitude coordinates recorded by Qualtrics during participants’ pre-scan surveys to reverse geocode their current zip code using an online batch geocoder (https://csv2geo.com). Two participants’ current zip codes could not be identified using this procedure; for these participants, their population density score was calculated using only their childhood values. For the remaining 52 participants, population density across all four life periods were averaged to compute a “lifetime density” score. Lifetime density did not significantly correlate with lifetime interracial contact (*r*(52) = 0.105, *p* = 0.450, 95% CI = [-0.167, 0.363]). We controlled for lifetime density in all of the analyses we report. In no case were the contact effects changed by the inclusion of lifetime density (if anything, the reported effects were stronger after accounting for density). It is therefore unlikely that exposure to more faces in general (i.e., higher lifetime population density) can explain the effects of contact that we report.

**ROI Results Including Population Density.**

In the following table (Supplementary Table 1) we report results of all ROI analyses including lifetime population density as a factor in the model. Importantly, in bilateral TPJ, DMPFC, and bilateral STS, no effects involving contact changed after including lifetime population density.

**Supplementary Table 3.** ROI analysis results including lifetime population density. L = left and R = right. Significant results are marked with an asterisk, *p* < 0.05.

| **Predictors** | ***B*** | ***SE*** | **df** | **95% CI** | ***t-*value** | ***p*-value** |
| --- | --- | --- | --- | --- | --- | --- |
| *L. TPJ* | | | | | | |
| (Intercept) | -1.085 | 0.119 | 50 | [-1.318, -0.852] | -9.133 | < 0.001 * |
| Target race | 0.049 | 0.105 | 150 | [-0.157, 0.254] | 0.463 | 0.644 |
| Task | 0.651 | 0.105 | 150 | [0.445, 0.856] | 6.197 | < 0.001 * |
| Lifetime contact | 0.035 | 0.120 | 50 | [-0.201, 0.271] | 0.292 | 0.772 |
| Lifetime population density | 0.093 | 0.119 | 50 | [-0.141, 0.326] | 0.776 | 0.441 |
| Target race x task | -0.120 | 0.210 | 150 | [-0.531, 0.292] | -0.571 | 0.569 |
| Target race x lifetime contact | -0.007 | 0.106 | 150 | [-0.215, 0.202] | -0.063 | 0.95 |
| Task x lifetime contact | -0.115 | 0.106 | 150 | [-0.323, 0.093] | -1.083 | 0.281 |
| Target race x lifetime population density | -0.059 | 0.105 | 150 | [-0.266, 0.147] | -0.563 | 0.574 |
| Task x lifetime population density | -0.002 | 0.105 | 150 | [-0.209, 0.204] | -0.022 | 0.983 |
| Lifetime contact x lifetime population density | -0.094 | 0.124 | 50 | [-0.337, 0.149] | -0.758 | 0.452 |
| Target race x task x lifetime contact | 0.064 | 0.213 | 150 | [-0.353, 0.480] | 0.301 | 0.764 |
| Target race x task x lifetime population density | -0.024 | 0.211 | 150 | [-0.438, 0.389] | -0.115 | 0.909 |
| Target race x lifetime contact x lifetime population density | -0.028 | 0.109 | 150 | [-0.243, 0.186] | -0.26 | 0.796 |
| Task x lifetime contact x lifetime population density | -0.087 | 0.109 | 150 | [-0.301, 0.128] | -0.791 | 0.43 |
| Target race x task x lifetime contact x lifetime population density | 0.1267 | 0.219 | 150 | [-0.302, 0.556] | 0.579 | 0.563 |
| *R. TPJ* | | | | | | |
| (Intercept) | -0.853 | 0.097 | 50 | [-1.043, -0.664] | -8.838 | < 0.001 * |
| Target race | -0.018 | 0.088 | 150 | [-0.190, 0.153] | -0.211 | 0.833 |
| Task | 0.761 | 0.088 | 150 | [0.589, 0.933] | 8.686 | < 0.001 * |
| Lifetime contact | 0.061 | 0.098 | 50 | [-0.131, 0.252] | 0.619 | 0.539 |
| Lifetime population density | 0.146 | 0.097 | 50 | [-0.044, 0.336] | 1.508 | 0.138 |
| Target race x task | -0.018 | 0.175 | 150 | [-0.361, 0.326] | -0.101 | 0.920 |
| Target race x lifetime contact | -0.014 | 0.089 | 150 | [-0.188, 0.160] | -0.156 | 0.876 |
| Task x lifetime contact | -0.187 | 0.089 | 150 | [-0.361, -0.014] | -2.114 | 0.036 * |
| Target race x lifetime population density | -0.098 | 0.088 | 150 | [-0.271, 0.074] | -1.117 | 0.266 |
| Task x lifetime population density | -0.053 | 0.088 | 150 | [-0.225, 0.120] | -0.600 | 0.550 |
| Lifetime contact x lifetime population density | 0.048 | 0.101 | 50 | [-0.149, 0.246] | 0.479 | 0.634 |
| Target race x task x lifetime contact | 0.083 | 0.177 | 150 | [-0.265, 0.430] | 0.467 | 0.641 |
| Target race x task x lifetime population density | 0.070 | 0.176 | 150 | [-0.275, 0.415] | 0.399 | 0.690 |
| Target race x lifetime contact x lifetime population density | -0.106 | 0.091 | 150 | [-0.285, 0.073] | -1.163 | 0.247 |
| Task x lifetime contact x lifetime population density | 0.165 | 0.091 | 150 | [-0.014, 0.344] | 1.811 | 0.072 |
| Target race x task x lifetime contact x lifetime population density | 0.129 | 0.183 | 150 | [-0.229, 0.487[ | 0.704 | 0.483 |
| *DMPFC* | | | | | | |
| (Intercept) | -0.627 | 0.163 | 50 | [-0.946, -0.309] | -3.857 | < 0.001 * |
| Target race | -0.327 | 0.155 | 150 | [-0.631, -0.024] | -2.115 | 0.036 * |
| Task | 0.571 | 0.155 | 150 | [0.268, 0.875] | 3.690 | < 0.001 * |
| Lifetime contact | 0.015 | 0.165 | 50 | [-0.308, 0.337] | 0.089 | 0.929 |
| Lifetime population density | 0.302 | 0.163 | 50 | [-0.018, 0.623] | 1.851 | 0.070 |
| Target race x task | -0.277 | 0.310 | 150 | [-0.884, 0.329] | -0.896 | 0.372 |
| Target race x lifetime contact | -0.010 | 0.157 | 150 | [-0.317, 0.297] | -0.063 | 0.950 |
| Task x lifetime contact | -0.583 | 0.157 | 150 | [-0.890, -0.276] | -3.722 | < 0.001 * |
| Target race x lifetime population density | -0.133 | 0.155 | 150 | [-0.438, 0.172] | -0.855 | 0.394 |
| Task x lifetime population density | 0.453 | 0.155 | 150 | [0.148, 0.758] | 2.913 | 0.004 * |
| Lifetime contact x lifetime population density | -0.244 | 0.170 | 50 | [-0.576, 0.088] | -1.439 | 0.156 |
| Target race x task x lifetime contact | -0.107 | 0.313 | 150 | [-0.721, 0.508] | -0.340 | 0.734 |
| Target race x task x lifetime population density | 0.135 | 0.311 | 150 | [-0.474, 0.745] | 0.435 | 0.664 |
| Target race x lifetime contact x lifetime population density | 0.011 | 0.161 | 150 | [-0.305, 0.328] | 0.070 | 0.945 |
| Task x lifetime contact x lifetime population density | -0.239 | 0.161 | 150 | [-0.555, 0.078] | -1.479 | 0.141 |
| Target race x task x lifetime contact x lifetime population density | -0.030 | 0.323 | 150 | [-0.663, 0.603] | -0.093 | 0.926 |
| *L. STS* | | | | | | |
| (Intercept) | -0.913 | 0.092 | 50 | [-1.093, -0.733] | -9.931 | < 0.001 * |
| Target race | -0.065 | 0.101 | 150 | [-0.264, 0.133] | -0.645 | 0.520 |
| Task | 0.422 | 0.101 | 150 | [0.224, 0.621] | 4.172 | < 0.001 * |
| Lifetime contact | -0.133 | 0.093 | 50 | [-0.316, 0.049] | -1.434 | 0.158 |
| Lifetime population density | 0.130 | 0.092 | 50 | [-0.051, 0.311] | 1.405 | 0.166 |
| Target race x task | -0.379 | 0.202 | 150 | [-0.776, 0.018] | -1.873 | 0.063 |
| Target race x lifetime contact | -0.003 | 0.102 | 150 | [-0.204, 0.198] | -0.028 | 0.977 |
| Task x lifetime contact | -0.132 | 0.102 | 150 | [-0.332, 0.069] | -1.285 | 0.201 |
| Target race x lifetime population density | -0.019 | 0.102 | 150 | [-0.218, 0.181] | -0.182 | 0.856 |
| Task x lifetime population density | 0.006 | 0.102 | 150 | [-0.193, 0.205] | 0.062 | 0.951 |
| Lifetime contact x lifetime population density | -0.001 | 0.096 | 50 | [-0.188, 0.187] | -0.006 | 0.995 |
| Target race x task x lifetime contact | 0.014 | 0.205 | 150 | [-0.388, 0.415] | 0.067 | 0.947 |
| Target race x task x lifetime population density | -0.162 | 0.203 | 150 | [-0.560, 0.236] | -0.797 | 0.427 |
| Target race x lifetime contact x lifetime population density | -0.069 | 0.106 | 150 | [-0.276, 0.138] | -0.654 | 0.514 |
| Task x lifetime contact x lifetime population density | 0.007 | 0.106 | 150 | [-0.200, 0.213] | 0.062 | 0.950 |
| Target race x task x lifetime contact x lifetime population density | 0.275 | 0.211 | 150 | [-0.138, 0.689] | 1.304 | 0.194 |
| *R. STS* | | | | | | |
| (Intercept) | -1.065 | 0.082 | 50 | [-1.226, -0.904] | -12.956 | < 0.001 * |
| Target race | 0.006 | 0.108 | 150 | [-0.205, 0.217] | 0.055 | 0.956 |
| Task | 0.662 | 0.108 | 150 | [0.451, 0.873] | 6.145 | < 0.001 * |
| Lifetime contact | 0.076 | 0.083 | 50 | [-0.087, 0.239] | 0.909 | 0.368 |
| Lifetime population density | 0.251 | 0.083 | 50 | [0.090, 0.413] | 3.046 | 0.004 * |
| Target race x task | -0.201 | 0.215 | 150 | [-0.623, 0.221] | -0.932 | 0.353 |
| Target race x lifetime contact | -0.041 | 0.109 | 150 | [-0.255, 0.173] | -0.376 | 0.707 |
| Task x lifetime contact | -0.197 | 0.109 | 150 | [-0.411, 0.016] | -1.810 | 0.072 |
| Target race x lifetime population density | -0.045 | 0.108 | 150 | [-0.257, 0.167] | -0.418 | 0.676 |
| Task x lifetime population density | 0.032 | 0.108 | 150 | [-0.180, 0.244] | 0.296 | 0.767 |
| Lifetime contact x lifetime population density | 0.053 | 0.086 | 50 | [-0.115, 0.221] | 0.616 | 0.541 |
| Target race x task x lifetime contact | -0.158 | 0.218 | 150 | [-0.585, 0.269] | -0.724 | 0.470 |
| Target race x task x lifetime population density | -0.068 | 0.216 | 150 | [-0.492, 0.356] | -0.316 | 0.752 |
| Target race x lifetime contact x lifetime population density | -0.013 | 0.112 | 150 | [-0.233, 0.207] | -0.119 | 0.906 |
| Task x lifetime contact x lifetime population density | -0.029 | 0.112 | 150 | [-0.249, 0.191] | -0.260 | 0.795 |
| Target race x task x lifetime contact x lifetime population density | 0.131 | 0.225 | 150 | [-0.310, 0.571] | 0.581 | 0.562 |
| *L. amygdala* | | | | | | |
| (Intercept) | 0.078 | 0.098 | 50 | [-0.113, 0.270] | 0.799 | 0.428 |
| Target race | 0.025 | 0.111 | 150 | [-0.192, 0.241] | 0.223 | 0.824 |
| Task | 0.312 | 0.111 | 150 | [0.095, 0.528] | 2.821 | 0.005 * |
| Lifetime contact | -0.019 | 0.099 | 50 | [-0.213, 0.175] | -0.193 | 0.848 |
| Lifetime population density | 0.390 | 0.098 | 50 | [0.198, 0.583] | 3.973 | < 0.001 * |
| Target race x task | 0.071 | 0.221 | 150 | [-0.362, 0.505] | 0.323 | 0.747 |
| Target race x lifetime contact | -0.017 | 0.112 | 150 | [-0.236, 0.203] | -0.148 | 0.882 |
| Task x lifetime contact | -0.383 | 0.112 | 150 | [-0.602, -0.163] | -3.421 | 0.001 * |
| Target race x lifetime population density | -0.025 | 0.111 | 150 | [-0.243, 0.192] | -0.229 | 0.819 |
| Task x lifetime population density | -0.153 | 0.111 | 150 | [-0.371, 0.064] | -1.380 | 0.170 |
| Lifetime contact x lifetime population density | -0.038 | 0.102 | 50 | [-0.238, 0.162] | -0.370 | 0.713 |
| Target race x task x lifetime contact | 0.250 | 0.224 | 150 | [-0.188, 0.688] | 1.117 | 0.266 |
| Target race x task x lifetime population density | -0.166 | 0.222 | 150 | [-0.601, 0.269] | -0.746 | 0.457 |
| Target race x lifetime contact x lifetime population density | -0.075 | 0.115 | 150 | [-0.301, 0.151] | -0.652 | 0.516 |
| Task x lifetime contact x lifetime population density | -0.094 | 0.115 | 150 | [-0.320, 0.131] | -0.820 | 0.414 |
| Target race x task x lifetime contact x lifetime population density | 0.538 | 0.230 | 150 | [0.086, 0.990] | 2.335 | 0.021 * |
| *R. amygdala* | | | | | | |
| (Intercept) | 0.517 | 0.123 | 50 | [0.276, 0.759] | 4.197 | < 0.001 * |
| Target race | 0.044 | 0.124 | 150 | [-0.199, 0.286] | 0.353 | 0.725 |
| Task | 0.243 | 0.124 | 150 | [0.001, 0.486] | 1.971 | 0.051 |
| Lifetime contact | -0.133 | 0.125 | 50 | [-0.378, 0.111] | -1.070 | 0.290 |
| Lifetime population density | 0.278 | 0.124 | 50 | [0.035, 0.520] | 2.243 | 0.029 * |
| Target race x task | 0.077 | 0.247 | 150 | [-0.407, 0.562] | 0.314 | 0.754 |
| Target race x lifetime contact | -0.144 | 0.125 | 150 | [-0.389, 0.101] | -1.151 | 0.251 |
| Task x lifetime contact | -0.051 | 0.125 | 150 | [-0.296, 0.194] | -0.406 | 0.685 |
| Target race x lifetime population density | 0.028 | 0.124 | 150 | [-0.215, 0.271] | 0.226 | 0.822 |
| Task x lifetime population density | -0.157 | 0.124 | 150 | [-0.401, 0.086] | -1.269 | 0.206 |
| Lifetime contact x lifetime population density | 0.050 | 0.129 | 50 | [-0.202, 0.302] | 0.388 | 0.699 |
| Target race x task x lifetime contact | 0.125 | 0.250 | 150 | [-0.365, 0.615] | 0.500 | 0.618 |
| Target race x task x lifetime population density | -0.259 | 0.248 | 150 | [-0.745, 0.227] | -1.044 | 0.298 |
| Target race x lifetime contact x lifetime population density | -0.150 | 0.129 | 150 | [-0.402, 0.102] | -1.164 | 0.246 |
| Task x lifetime contact x lifetime population density | 0.128 | 0.129 | 150 | [-0.124, 0.380] | 0.994 | 0.322 |
| Target race x task x lifetime contact x lifetime population density | 0.538 | 0.258 | 150 | [0.033, 1.043] | 2.088 | 0.038 * |

Although the effects of contact were unchanged, there was a significant interaction between lifetime population density and task in the DMPFC (Supplementary Table 3; see Supplementary Fig. 3). For low lifetime population density, there was no significant difference in DMPFC BOLD activity depending on task (*B* = -0.335, *SE* = 0.346, *df* = 150, 95% CI = [-1.013, 0.344], *t*-value = -0.966, *p*-value =0.336); however, there was greater DMPFC activity during the social judgment condition than the non-social judgment condition among average (*B* = 0.571, *SE* = 0.155, *df* = 150, 95% CI = [0.268, 0.875], *t*-value = 3.690, *p*-value < 0.001) and high (*B* = 1.477, *SE* = 0.348, *df* = 150, 95% CI = [0.794, 2.160], *t*-value = 4.239, *p* < 0.001) population density perceivers. As population density increased, DMPFC activity during the social judgment condition significantly increased (*B* = 0.529, *SE* = 0.181, *df* = 73.950, 95% CI = [0.174, 0.883], *t*-value = 2.923, *p*-value = 0.005), whereas DMPFC activity during the non-social judgment condition did not change significantly (*B* = 0.076, *SE* = 0.181, *df* = 73.950, 95% CI = [-0.279, 0.431], *t*-value = 0.420, *p*-value = 0.676).

**Supplementary Fig. 3.** The two-way interaction of task and lifetime population density significantly predicted BOLD activity in DMPFC. Asterisks denote simple significant differences (*p* < 0.05). Crosses denote significant slopes (*p* < 0.05).

Similarly, in both left and right amygdalae, although the effects of contact from prior to the inclusion of population density remained unchanged, there was also a significant 4-way interaction between task, target race, lifetime contact, and lifetime population density. The present study was not powered to decompose this 4-way interaction; therefore, we limit our interpretation of these results. However, we present the simple differences and slopes for these interactions for interested readers below.

**Left amygdala four-way interaction.** For low lifetime density (re-centered at -2SD) perceivers viewing Black targets, there were no significant simple differences or slopes involving contact and task (social vs. non-social for low density/low contact/Black targets: *B* = -0.022, *SE* = 0.758, *df* = 150, 95% CI = [-1.507, 1.463], *t*-value = -0.029, *p*-value = 0.977; social vs. non-social for low density/average contact/Black targets: *B* = 0.417, *SE* = 0.350, *df* = 150, 95% CI = [-0.268, 1.102], *t*-value = 1.192, *p*-value = 0.235; social vs. non-social for low density/high contact/Black targets: *B* = 0.856, *SE* = 0.777, *df* = 150, 95% CI = [-0.667, 2.378], *t*-value = 1.102, *p*-value = 0.272; contact slope for low density/social/Black targets: *B* = 0.099, *SE* = 0.299, *df* = 146.810, 95% CI = [-0.487, 0.685], *t*-value = 0.332, *p*-value = 0.740; contact slope for low density/non-social/Black targets: *B* = -0.120, *SE* = 0.299, *df* = 146.810, 95% CI = [-0.706, 0.466], *t*-value = -0.402, *p­*-value = 0.688). However, for White targets, there was significantly greater activity in the social condition than in the non-social condition for low (*B* = 2.033, *SE* = 0.757, *df* = 150, 95% CI = [0.548, 3.518], *t*-value = 2.685, *p*-value = 0.008) and average (*B* = 0.819, *SE* = 0.350, *df* = 150, 95% CI = [0.134, 1.505], *t*-value = 2.344, *p*-value = 0.020) but not high (*B* = -0.394, *SE* = 0.777, *df* = 150, 95% CI = [-1.916, 1.128], *t*-value = -0.507, *p*-value = 0.613) contact perceivers. Both the slopes for contact in the social judgment condition with White targets (*B* = -0.180, *SE* = 0.299, *df* = 146.810, 95% CI = [-0.766, 0.406], *t*-value = -0.603, *p*-value = 0.547) and the non-social judgment condition with White targets (*B* = 0.427, *SE* = 0.299, *df* = 146.810, 95% CI = [-0.159, 1.012], *t*-value = 1.427, *p*-value = 0.156) were not significant.

For average lifetime density, low contact perceivers viewing Black targets showed greater left amygdala activity in the social judgment condition than in the non-social judgment condition (*B* = 1.291, *SE* = 0.355, *df* = 150, 95% CI = [0.595, 1.987], *t*-value = 3.637, *p*-value < 0.001). Average contact perceivers viewing Black targets did not have different levels of left amygdala activity for each task (*B* = 0.276, *SE* = 0.156, *df* = 150, 95% CI = [-0.030, 0.582], *t*-value = 1.767, *p*-value = 0.079). High contact perceivers viewing Black targets showed greater left amygdala activity in the non-social condition than in the social condition (*B* = -0.739, *SE* = 0.351, *df* = 150, 95% CI = [-1.426, -0.052], *t*-value = -2.107, *p*-value = 0.037). The slopes for contact in the social condition with Black targets (*B* = -0.265, *SE* = 0.138, *df* = 146.810, 95% CI = [-0.536, 0.007], *t*-value = -1.911, *p*-value = 0.058) and in the non-social condition with Black targets (*B* = 0.243, *SE* = 0.138, *df* = 146.810, 95% CI = [-0.028, 0.514], *t*-value = 1.755, *p*-value = 0.081) were not significant. When viewing White targets, average lifetime density perceivers with low (*B* = 0.863, *SE* = 0.355, *df* = 150, 95% CI = [0.167, 1.559], *t*-value = 2.430, *p*-value = 0.016) and average (*B* = 0.347, *SE* = 0.156, *df* = 150, 95% CI = [0.041, 0.654], *t*-value = 2.223, *p*-value = 0.028) contact showed greater left amygdala activity in the social condition than in the non-social condition. High contact perceivers viewing White targets did not show different levels of left amygdala activity regardless of whether they were performing the social or non-social task (*B* = -0.168, *SE* = 0.351, *df* = 150, 95% CI = [-0.855, 0.520], *t*-value = -0.478, *p*-value = 0.633). Again, the slopes for contact in the social judgment condition with White targets (*B* = -0.156, *SE* = 0.138, *df* = 146.810, 95% CI = [-0.428, 0.115], *t*-value = -1.128, *p*-value = 0.261) and in the non-social judgment condition with White targets (*B* = 0.101, *SE* = 0.138, *df* = 146.810, 95% CI = [-0.170, 0.373], *t*-value = 0.732, *p*-value = 0.465) were not significant.

For high lifetime density, low contact perceivers viewing Black targets showed greater left amygdala activity in the social judgment condition than in the non-social judgment condition (*B* = 2.608, *SE* = 0.884, *df* = 150, 95% CI = [0.871, 4.338], *t*-value = 2.945, *p*-value = 0.004). Average contact perceivers viewing Black targets did not have different levels of left amygdala activity for each task (*B* = 0.136, *SE* = 0.352, *df* = 150, 95% CI = [-0.554, 0.825], *t*-value = 0.385, *p*-value = 0.701). High contact perceivers viewing Black targets showed greater left amygdala activity in the non-social judgment condition than in the social judgment condition (*B* = -2.334, *SE* = 0.795, *df* = 150, 95% CI = [-3.892, -0.775], *t*-value = -2.935, *p*-value = 0.004). The slopes for contact in the social judgment condition with Black targets (*B* = -0.628, *SE* = 0.334, *df* = 146.810, 95% CI = [-1.284, 0.027], *t*-value = -1.880, *p*-value = 0.062) and in the non-social judgment condition with Black targets (*B* = 0.606, *SE* = 0.334, *df* = 146.810, 95% CI = [-0.049, 1.261], *t*-value = 1.813, *p*-value = 0.072) were not significant. For White targets, there were no significant simple differences or slopes involving contact and task (social vs. non-social for high density/low contact/White targets: *B* = -0.308, *SE* = 0.884, *df* = 150, 95% CI = [-2.041, 1.426], *t*-value = -0.348, *p*-value = 0.729; social vs. non-social for high density/average contact/White targets: *B* = -0.125, *SE* = 0.352, *df* = 150, 95% CI = [-0.814, 0.565], *t*-value = -0.354, *p*-value = 0.724; social vs. non-social for high density/high contact/White targets: *B* = 0.058, *SE* = 0.795, *df* = 150, 95% CI = [-1.500, 1.617], *t*-value = 0.073, *p*-value = 0.942; contact slope for high density/social/White targets: *B* = -0.132, *SE* = 0.334, *df* = 146.810, 95% CI = [-0.787, 0.523], *t*-value = -0.395, *p*-value = 0.693; contact slope for high density/non-social/White targets: *B* = -0.224, *SE* = 0.334, *df* = 146.810, 95% CI = [-0.879, 0.432], *t*-value = -0.669, *p­*-value = 0.504).

**Right amygdala four-way interaction.** The only significant simple differences from this interaction involved low and average contact perceivers with low levels of lifetime population density viewing White targets. For these perceivers, there was significantly greater right amygdala BOLD activity during the social judgment condition than the non-social judgment condition (low contact: *B* = 2.420, *SE* = 0.847, *df* = 150, 95% CI = [0.761, 4.080], *t*-value = 2.859, *p*-value = 0.005; average contact: *B* = 0.856, *SE* = 0.391, *df* = 150, 95% CI = [0.090, 1.622], *t*-value = 2.191, *p*-value = 0.030). For high contact low population density perceivers viewing White targets, the difference in right amygdala BOLD activity between the social judgment condition and the non-social judgment condition was not significant (*B* = -0.708, *SE* = 0.868, *df* = 150, 95% CI = [-2.409, 0.993], *t*-value = -0.816, *p*-value = 0.416). The slopes for contact among low density perceivers viewing White targets during the social (*B* = -0.546, *SE* = 0.357, *df* = 129.240, 95% CI = [-1.245, 0.152], *t*-value = -1.533, *p*-value = 0.128) and non-social (*B* = 0.236, *SE* = 0.357, *df* = 129.240, 95% CI = [-0.463, 0.934], *t*-value = 0.661, *p*-value = 0.510) conditions were not significant. For low lifetime density perceivers viewing Black targets, there were no significant simple differences or slopes involving contact and task (social vs. non-social for low density/low contact/Black targets: *B* = -0.077, *SE* = 0.847, *df* = 150, 95% CI = [-1.736, 1.583], *t*-value = -0.091, *p*-value = 0.928; social vs. non-social for low density/average contact/Black targets: *B* = 0.260, *SE* = 0.391, *df* = 150, 95% CI = [-0.505, 1.026], *t*-value = 0.666, *p*-value = 0.506; social vs. non-social for low density/high contact/Black targets: *B* = 0.598, *SE* = 0.868, *df* = 150, 95% CI = [-1.104, 2.299], *t*-value = 0.688, *p*-value = 0.492; contact slope for low density/social/Black targets: *B* = -0.227, *SE* = 0.357, *df* = 129.240, 95% CI = [-0.926, 0.472], *t*-value = -0.637, *p*-value = 0.526; contact slope for low density/non-social/Black targets: *B* = -0.396, *SE* = 0.357, *df* = 129.240, 95% CI = [-1.094, 0.303], *t*-value = -1.109, *p­*-value = 0.269).

For average lifetime density perceivers viewing Black targets, there were no significant simple differences or slopes involving contact and task (social vs. non-social for average density/low contact/Black targets: *B* = 0.431, *SE* = 0.397, *df* = 150, 95% CI = [-0.346, 1.209], *t*-value = 1.087, *p*-value = 0.279; social vs. non-social for average density/average contact/Black targets: *B* = 0.205, *SE* = 0.175, *df* = 150, 95% CI = [-0.138, 0.547], *t*-value = 1.172, *p*-value = 0.243; social vs. non-social for average density/high contact/Black targets: *B* = -0.022, *SE* = 0.392, *df* = 150, 95% CI = [-0.790, 0.746], *t*-value = -0.056, *p*-value = 0.956; contact slope for average density/social/Black targets: *B* = -0.118, *SE* = 0.165, *df* = 129.240, 95% CI = [-0.442, 0.206], *t*-value = -0.715, *p*-value = 0.476; contact slope for average density/non-social/Black targets: *B* = -0.005, *SE* = 0.165, *df* = 129.240, 95% CI = [-0.329, 0.319], *t*-value = -0.029, *p­*-value = 0.977). Similarly, for average lifetime density perceivers viewing White targets, there were no significant simple differences or slopes involving contact and task (social vs. non-social for average density/low contact/White targets: *B* = 0.259, *SE* = 0.397, *df* = 150, 95% CI = [-0.519, 1.036], *t*-value = 0.652, *p*-value = 0.515; social vs. non-social for average density/average contact/White targets: *B* = 0.282, *SE* = 0.175, *df* = 150, 95% CI = [-0.060, 0.625], *t*-value = 1.616, *p*-value = 0.108; social vs. non-social for average density/high contact/White targets: *B* = 0.306, *SE* = 0.392, *df* = 150, 95% CI = [-0.463, 1.074], *t*-value = 0.780, *p*-value = 0.437; contact slope for average density/social/White targets: *B* = -0.200, *SE* = 0.165, *df* = 129.240, 95% CI = [-0.523, 0.124], *t*-value = -1.208, *p*-value = 0.229; contact slope for average density/non-social/White targets: *B* = -0.211, *SE* = 0.165, *df* = 129.240, 95% CI = [-0.535, 0.112], *t*-value = -1.279, *p­*-value = 0.203).

For high lifetime density perceivers viewing Black targets, there were no significant simple differences or slopes involving contact and task (social vs. non-social for high density/low contact/Black targets: *B* = 0.940, *SE* = 0.988, *df* = 150, 95% CI = [-0.998, 2.877], *t*-value = 0.951, *p*-value = 0.343; social vs. non-social for high density/average contact/Black targets: *B* = 0.149, *SE* = 0.393, *df* = 150, 95% CI = [-0.621, 0.920], *t*-value = 0.379, *p*-value = 0.705; social vs. non-social for high density/high contact/Black targets: *B* = -0.642, *SE* = 0.889, *df* = 150, 95% CI = [-2.383, 1.101], *t*-value = -0.722, *p*-value = 0.472; contact slope for high density/social/Black targets: *B* = -0.009, *SE* = 0.399, *df* = 129.240, 95% CI = [-0.791, 0.772], *t*-value = -0.024, *p*-value = 0.981; contact slope for high density/non-social/Black targets: *B* = 0.386, *SE* = 0.399, *df* = 129.240, 95% CI = [-0.396, 1.168], *t*-value = 0.968, *p­*-value = 0.335). Similarly, for high lifetime density perceivers viewing White targets, there were no significant simple differences or slopes involving contact and task (social vs. non-social for high density/low contact/White targets: *B* = -1.903, *SE* = 0.988, *df* = 150, 95% CI = [-3.840, 0.034], *t*-value = -1.925, *p*-value = 0.056; social vs. non-social for high density/average contact/White targets: *B* = -0.292, *SE* = 0.393, *df* = 150, 95% CI = [-1.062, 0.479], *t*-value = -0.742, *p*-value = 0.459; social vs. non-social for high density/high contact/White targets: *B* = 1.319, *SE* = 0.889, *df* = 150, 95% CI = [-0.423, 3.061], *t*-value = 1.484, *p*-value = 0.140; contact slope for high density/social/White targets: *B* = 0.147, *SE* = 0.399, *df* = 129.240, 95% CI = [-0.634, 0.929], *t*-value = 0.369, *p*-value = 0.713; contact slope for high density/non-social/White targets: *B* = -0.658, *SE* = 0.399, *df* = 129.240, 95% CI = [-1.440, 0.123], *t*-value = -1.651, *p­*-value = 0.101).

**Supplementary Discussion 5. Behavioral Results.**

We analyzed social judgment trials and non-social judgment trials in separate models. During the social judgment trials, there was only a significant main effect of race such that Black faces were rated as appearing more interested than White faces (*B* = -0.294, *SE* = 0.041, *df* = 57.96, *t*-value = -7.204, *p* < 0.001, 95% CI = [-0.374, -0.214]). During the non-social judgment trials, there was a significant interaction between race and lifetime contact (*B* = -0.073, *SE* = 0.032, *df* = 60.47, *t*-value = -2.250, *p* = 0.028, 95% CI = [-0.136, -0.009]; see Supplementary Fig. 4). Among low (-2SD) and average (0SD) contact participants, there was no significant difference between symmetry ratings by race (low contact: *B* = 0.099, *SE* = 0.072, *df* = 59.35, *t*-value = 1.362, *p* = 0.179, 95% CI = [-0.043, 0.240]; average contact: *B* = -0.047, *SE* = 0.032, *df* = 59.53, *t*-value = -1.438, *p* = 0.156, 95% CI = [-0.110, 0.017]). However, among high contact participants, Black faces were rated as significantly more symmetrical than White faces (*B* = -0.192, *SE* = 0.072, *df* = 61.24, *t*-value = -2.665, *p* = 0.010, 95% CI = [-0.333, -0.051]). We also ran models with the Black and White conditions dummy coded to allow us to assess the effect of lifetime interracial contact on symmetry ratings within each race. There was no significant difference on symmetry ratings during Black trials as a function of contact (*B* = -0.027, *SE* = 0.040, *df* = 76, *t*-value = -0.684, *p* = 0.496, 95% CI = [-0.105, 0.051]). However, as lifetime contact increased, participants rated White faces as significantly less symmetrical (*B* = -0.100, *SE* = 0.040, *df* = 77, *t*-value = -2.2524, *p* = 0.014, 95% CI = [-0.178, -0.022]).

**Supplementary Fig. 4.** The interaction between lifetime contact and target race significantly predicted participants’ symmetry ratings in the non-social judgment task. Among low and average contact participants, symmetry ratings did not significantly differ by race; however, among high contact participants, Black faces were rated as significantly more symmetrical than White faces. Significant simple differences are denoted with an asterisk and significant slopes are denoted with a cross.

**Supplementary Discussion 6. PLS Results Tables.**

**Supplementary Table 4.** Results of task PLS analysis. BSRs are reported as absolute values.

| **L/R** | **BA** | **Region** | **Cluster size** | **MNI coordinates** | | | **BSR** |
| --- | --- | --- | --- | --- | --- | --- | --- |
|  |  |  |  | ***x*** | ***y*** | ***z*** |  |
| *Negative (i.e., interest > symmetry)* | | | | | | | |
| R | 21 | Middle temporal gyrus | 2222 | 57 | -3 | -27 | 6.43 |
| R |  | Temporoparietal junction/posterior superior temporal gyrus |  | 60 | -54 | 12 | 6.33 |
| R | 39 | Inferior temporal gyrus |  | 51 | -48 | 21 | 5.84 |
| R | 39 | Angular gyrus |  | 57 | -66 | 27 | 4.60 |
| R | 38 | Middle temporal pole |  | 42 | 15 | -39 | 3.70 |
| R | 38 | Superior temporal pole |  | 30 | 12 | -30 | 3.51 |
| L | 21 | Temporal pole | 1483 | -54 | -3 | -27 | 5.53 |
| L | 21 | Anterior superior temporal sulcus |  | -63 | -39 | -3 | 5.30 |
| L | 39 | Temporoparietal junction |  | -60 | -45 | 21 | 4.88 |
| L | 21 | Middle temporal gyrus |  | -54 | -12 | -15 | 4.83 |
| L | 39 | Posterior superior temporal sulcus |  | -51 | -60 | 15 | 4.59 |
| R | 23 | Posterior cingulate gyrus | 819 | 9 | -48 | 30 | 5.49 |
| L | 31 | Precuneus |  | -6 | -60 | 27 | 5.32 |
| R | 31 | Precuneus |  | 9 | -54 | 33 | 5.00 |
| R | 47 | Inferior frontal gyrus | 204 | 51 | 27 | -3 | 5.04 |
| L |  | Cerebellum | 162 | -24 | -84 | -45 | 4.80 |
| L | 47 | Inferior frontal gyrus | 263 | -48 | 27 | -6 | 4.35 |
| L | 6 | Precentral gyrus | 232 | -39 | 9 | 48 | 4.21 |
| L | 38 | Superior temporal pole | 46 | -30 | 12 | -30 | 4.12 |
| R | 8 | Precentral gyrus | 95 | 39 | 9 | 42 | 3.80 |
| R | 6 | Superior frontal gyrus | 255 | 12 | 30 | 60 | 3.79 |
|  | 10 | Dorsomedial prefrontal cortex | 183 | 9 | 57 | 21 | 3.72 |
| R |  | Cerebellum | 67 | 48 | -84 | -42 | 3.46 |
|  | 10 | Ventromedial prefrontal cortex | 39 | -12 | 42 | -6 | 3.38 |
|  | 9 | Dorsomedial prefrontal cortex | 21 | -9 | 54 | 33 | 3.04 |
| *Positive (i.e., symmetry > interest)* | | | | | | | |
| R | 19 | Middle occipital gyrus | 3787 | 36 | -84 | 12 | 8.95 |
| R | 7 | Superior parietal lobule |  | 30 | -60 | 54 | 7.63 |
| R |  | Fusiform gyrus |  | 51 | -54 | -12 | 6.62 |
| R | 40 | Supramarginal gyrus |  | 48 | -33 | 39 | 6.50 |
| R | 6 | Postcentral gyrus |  | 51 | 6 | 27 | 6.42 |
| R | 40 | Inferior parietal lobule |  | 42 | -36 | 39 | 6.29 |
| L |  | Inferior temporal gyrus | 2459 | -54 | -60 | -12 | 8.32 |
| L | 19 | Middle occipital gyrus |  | -33 | -90 | 21 | 7.06 |
| L | 7 | Superior parietal lobule |  | -21 | -66 | 51 | 6.90 |
| L |  | Middle occipital gyrus |  | -39 | -87 | -3 | 5.85 |
| L |  | Fusiform gyrus |  | -36 | -48 | -15 | 4.06 |
| L | 7 | Inferior parietal lobule |  | -42 | -45 | 51 | 4.72 |
| R | 6 | Posterior middle frontal gyrus | 292 | 30 | 0 | 54 | 5.59 |
| R |  | Insula | 62 | 36 | 18 | 3 | 4.75 |
| R | 46 | Middle frontal gyrus | 89 | 45 | 39 | 9 | 4.60 |
| L | 6 | Posterior middle frontal gyrus | 93 | -27 | -6 | 51 | 3.73 |

**Supplementary Table 5.** Results of behavioral PLS analysis, first LV. BSRs are reported as absolute values.

| **L/R** | **BA** | **Region** | **Cluster size** | **MNI coordinates** | | | **BSR** |
| --- | --- | --- | --- | --- | --- | --- | --- |
|  |  |  |  | ***x*** | ***y*** | ***z*** |  |
| *Positive; decreased co-activation with increasing contact during symmetry trials* | | | | | | | |
| None. | | | | | | | |
| *Negative; increased co-activation with increasing contact during symmetry trials* | | | | | | | |
| L |  | Cerebellum | 14048 | -30 | -69 | -45 | 7.22 |
|  | 32 | Middle anterior cingulate cortex |  | 3 | 18 | 33 | 5.90 |
| L |  | Inferior temporal gyrus/fusiform gyrus |  | -51 | -42 | -18 | 5.81 |
| L |  | Putamen |  | -18 | 12 | 0 | 5.67 |
|  |  | Brainstem |  | 6 | -30 | -33 | 5.11 |
| L |  | Postcentral gyrus |  | -60 | -18 | 27 | 4.80 |
|  |  | Thalamus |  | 6 | -9 | 9 | 4.80 |
| L |  | Middle frontal gyrus |  | -27 | 48 | 33 | 4.66 |
| L |  | Precuneus |  | -18 | -60 | 33 | 4.49 |
|  | 8 | Dorsomedial prefrontal cortex |  | 6 | 39 | 36 | 4.45 |
| R |  | Caudate nucleus |  | 18 | 18 | 0 | 4.43 |
| L | 47 | Anterior insula/middle frontal gyrus |  | -33 | 30 | -6 | 4.36 |
| L |  | Middle anterior cingulate cortex |  | -18 | -21 | 36 | 4.10 |
| R | 8 | Middle frontal gyrus |  | 36 | 18 | 36 | 4.02 |
| R | 21 | Posterior superior temporal sulcus |  | 48 | -36 | -9 | 4.02 |
| L |  | Insula |  | -39 | 0 | 3 | 4.00 |
| R | 20 | Inferior temporal gyrus |  | 48 | -15 | -30 | 3.94 |
| L |  | Caudate nucleus |  | -12 | 24 | 3 | 3.61 |
| R |  | Fusiform gyrus |  | 39 | -24 | -24 | 3.53 |
| L |  | Cuneus |  | -24 | -75 | 18 | 3.47 |
|  | 9 | Anterior cingulate cortex |  | 3 | 39 | 21 | 3.46 |
| L |  | Inferior parietal lobule |  | -54 | -16 | 27 | 3.40 |
| R |  | Cuneus |  | 15 | -75 | 21 | 3.32 |
| R | 38 | Temporal pole |  | 27 | 15 | -42 | 3.18 |
| R | 47 | Anterior insula/middle frontal gyrus |  | 33 | 30 | -6 | 3.03 |
| L |  | Anterior fusiform gyrus |  | -36 | -27 | -24 | 2.93 |
| R | 11 | Orbitofrontal cortex |  | 24 | 39 | -15 | 2.86 |
| R | 7 | Superior parietal lobule | 237 | 36 | -60 | 63 | 4.96 |
|  |  | Paracentral lobule | 437 | 0 | -39 | 75 | 4.53 |
| L | 7 | Precuneus |  | -6 | -57 | 57 | 3.17 |
| L | 47 | Inferior frontal gyrus | 41 | -33 | 30 | -6 | 4.36 |
| L |  | Anterior superior temporal sulcus | 115 | -42 | -3 | -24 | 4.28 |
| L |  | Amygdala |  | -24 | -3 | -24 | 2.91 |
| L | 46 | Inferior frontal gyrus | 58 | -42 | 33 | 15 | 4.14 |
| L | 38 | Temporal pole | 94 | -51 | 12 | -36 | 3.73 |
| R |  | Postcentral gyrus | 35 | 30 | -30 | 45 | 3.66 |
| L | 10 | Anterior prefrontal cortex | 27 | -21 | 51 | 9 | 3.32 |

**Supplementary Table 6.** Results of behavioral PLS analysis, second LV.

| **L/R** | **BA** | **Region** | **Cluster size** | **MNI coordinates** | | | **BSR** |
| --- | --- | --- | --- | --- | --- | --- | --- |
|  |  |  |  | ***x*** | ***y*** | ***z*** |  |
| *Positive; decreased co-activation with increasing contact* | | | | | | | |
| R |  | Parahippocampal gyrus | 63 | 27 | -21 | -30 | 4.22 |
| R |  | Anterior fusiform gyrus |  | 39 | -21 | -30 | 4.21 |
|  | 11 | Medial orbitofrontal cortex | 21 | 0 | 24 | -21 | 3.57 |
|  | 10 | Ventromedial prefrontal cortex | 32 | -15 | 51 | 0 | 3.95 |
| R | 19 | Inferior occipital cortex | 110 | 45 | -84 | -3 | 3.94 |
| R | 6 | Middle frontal gyrus | 502 | 42 | -6 | 54 | 3.91 |
|  |  | Postcentral gyrus |  | 39 | -21 | 50 | 3.27 |
|  |  | Ventromedial prefrontal cortex | 29 | 18 | 45 | 6 | 3.54 |
| L | 10 | Middle frontal gyrus | 65 | -36 | 57 | 3 | 3.48 |
| R | 6 | Rolandic operculum | 87 | 57 | 6 | 9 | 3.46 |
| R |  | Superior occipital gyrus | 56 | 21 | -90 | 15 | 3.18 |
| *Negative; increased co-activation with increasing contact* | | | | | | | |
|  |  | Brainstem | 174 | 0 | -30 | -18 | 4.30 |
| R |  | Superior occipital gyrus | 80 | 21 | -60 | 36 | 4.06 |

**Supplemental References**

Allan, S., & Gilbert, P. (1995). A social comparison scale: Psychometric properties and relationship to psychopathology. In *Personality and Individual Differences* (Vol. 19). https://doi.org/10.1016/0191-8869(95)00086-L

Anderson, C., John, O. P., & Keltner, D. (2011). The personal sense of power. *Journal of Personality*, *80*, 313–344. https://doi.org/10.1111/j.1467-6494.2011.00734.x

Carriere, J. S. A., Seli, P., & Smilek, D. (2013). Wandering in both mind and body: Individual differences in mind wandering and inattention predict fidgeting. In *Canadian Journal of Experimental Psychology/Revue canadienne de psychologie expérimentale* (Vol. 67, pp. 19–31). Educational Publishing Foundation. https://doi.org/10.1037/a0031438

Carver, C. S., & White, T. L. (1994). Behavioral inhibition, behavioral activation, and affective responses to impending reward and punishment: The BIS/BAS scales. In *Journal of Personality and Social Psychology* (Vol. 67, Issue 2, pp. 319–333). American Psychological Association. https://doi.org/10.1037/0022-3514.67.2.319

Connor, K. M., & Davidson, J. R. T. (2003). Development of a new resilience scale: The Connor-Davidson Resilience Scale (CD-RISC). *Depression and Anxiety*, *18*, 76–82. https://doi.org/10.1002/da.10113

Duchaine, B., & Nakayama, K. (2006). The Cambridge Face Memory Test: Results for neurologically intact individuals and an investigation of its validity using inverted face stimuli and prosopagnosic participants. *Neuropsychologia*, *44*, 576–585. https://doi.org/10.1016/j.neuropsychologia.2005.07.001

Greenwald, A. G., McGhee, D. E., & Schwartz, J. L. K. (1998). Measuring individual differences in implicit cognition: The implicit association test. *Journal of Personality and Social Psychology*, *74*, 1464–1480. https://doi.org/10.1037/0022-3514.74.6.1464

Greenwald, A. G., Nosek, B. A., & Banaji, M. R. (2003). Understanding and using the Implicit Association Test: I. An improved scoring algorithm. In *Journal of Personality and Social Psychology* (Vol. 85, pp. 197–216). American Psychological Association. https://doi.org/10.1037/0022-3514.85.2.197

Heider, F., & Simmel, M. (1944). An experimental study of apparent behavior. *The American Journal of Psychology*, *57*, 243–259. https://doi.org/10.2307/1416950

McConahay, J. B. (1986). Modern racism, ambivalence, and the Modern Racism Scale. In J. F. Dovidio & S. L. Gaertner (Eds.), *Prejudice, discrimination, and racism* (pp. 91–125). Academic Press.

Nicholson, N., Soane, E., Fenton‐O’Creevy, M., & Willman, P. (2005). Personality and domain‐specific risk taking. *Journal of Risk Research*, *8*(2), 157–176. https://doi.org/10.1080/1366987032000123856

Plant, E. A., & Devine, P. G. (1998). Internal and external motivation to respond without prejudice scales. *Journal of Personality and Social Psychology*, *75*, 3–4. https://doi.org/10.1037/t03881-000

Reniers, R. L. E. P., Corcoran, R., Drake, R., Shryane, N. M., & Völlm, B. A. (2011). The QCAE: A questionnaire of cognitive and affective empathy. *Journal of Personality Assessment*, *93*, 84–95. https://doi.org/10.1080/00223891.2010.528484

Rushton, J. P., Chrisjohn, R. D., & Fekken, G. C. (1981). The altruistic personality and the self-report altruism scale. *Personality and Individual Differences*, *2*, 293–302. https://doi.org/10.1016/0191-8869(81)90084-2

Russell, D., Peplau, L. A., & Cutrona, C. E. (1980). The revised UCLA Loneliness Scale: Concurrent and discriminant validity evidence. In *Journal of Personality and Social Psychology* (Vol. 39, pp. 472–480). American Psychological Association. https://doi.org/10.1037/0022-3514.39.3.472

Schlegel, K., & Scherer, K. R. (2016). Introducing a short version of the Geneva Emotion Recognition Test (GERT-S): Psychometric properties and construct validation. *Behavior Research Methods*, *48*, 1383–1392. https://doi.org/10.3758/s13428-015-0646-4

Yamagishi, T., & Yamagishi, M. (1994). Trust and commitment in the United States and Japan. In *Motivation and Emotion* (Vol. 18, pp. 129–166). Springer. https://doi.org/10.1007/BF02249397
